# AMPK associates with and causes fragmentation of the Golgi by phosphorylating the guanine nucleotide exchange factor GBF1

**DOI:** 10.1242/jcs.262182

**Published:** 2024-12-23

**Authors:** Jordana B. Freemantle, Mhairi C. Towler, Emma R. Hudson, Thomas Macartney, Monika Zwirek, David J. K. Liu, David A. Pan, Sreenivasan Ponnambalam, D. Grahame Hardie

**Affiliations:** ^1^ Division of Cell Signalling & Immunology and School of Life Sciences, University of Dundee, Dundee DD1 5EH, Scotland, UK; ^2^ MRC Protein Phosphorylation and Ubiquitylation Unit, School of Life Sciences, University of Dundee, Dundee DD1 5EH, Scotland, UK; ^3^ Endothelial Cell Biology Unit, School of Molecular & Cellular Biology, University of Leeds, Leeds LS2 9JT, UK

**Keywords:** AMP-activated protein kinase, Subcellular localization, Golgi, Membrane trafficking, Protein secretion

## Abstract

AMP-activated protein kinase (AMPK) is an energy sensor that regulates cellular functions in response to changes in energy availability. However, whether AMPK activity is spatially regulated, and the implications for cell function, have been unclear. We now report that AMPK associates with the Golgi, and that its activation by two specific pharmacological activators leads to Golgi fragmentation similar to that caused by the antibiotic Golgicide A, an inhibitor of Golgi-specific Brefeldin A resistance factor-1 (GBF1), a guanine nucleotide exchange factor that targets ADP-ribosylation factor 1 (ARF1). Golgi fragmentation in response to AMPK activators is lost in cells carrying gene knockouts of AMPK-α subunits. AMPK has been previously reported to phosphorylate GBF1 at residue Thr1337, and its activation causes phosphorylation at that residue. Importantly, Golgi disassembly upon AMPK activation is blocked in cells expressing a non-phosphorylatable GBF1-T1337A mutant generated by gene editing. Furthermore, the trafficking of a plasma membrane-targeted protein through the Golgi complex is delayed by AMPK activation. Our findings provide a mechanism to link AMPK activation during cellular energy stress to downregulation of protein trafficking involving the Golgi.

## Teaser

**Summary:** The protein kinase AMPK displays Golgi localization and mediates GBF1 phosphorylation and Golgi fragmentation, thus linking cellular energy homeostasis to protein secretion.

## INTRODUCTION

AMP-activated protein kinase (AMPK) is a sensor of cellular energy and nutrient status that is expressed in almost all eukaryotic cells as stable complexes comprising catalytic α subunits and regulatory β and γ subunits (Gonzalez et al., 2020; Steinberg and Hardie, 2023; Trefts and Shaw, 2021). In humans there are multiple isoforms of each subunit (α1 and α2, β1 and β2, and γ1, γ2 and γ3) encoded by distinct genes (*PRKAA1*, *PRKAA2*, *PRKAB1*, *PRKAB2*, *PRKAG1*, *PRKAG2* and *PRKAG3*)*,* giving rise to up to 12 heterotrimeric combinations (Ross et al., 2016b). The classical (or canonical) activation mechanism for AMPK occurs in response to cellular energy stress, signalled by increases in AMP:ATP ratios. Binding of AMP to the crucial CBS3 site on the γ subunit, where it displaces ATP (Yan et al., 2021) activates AMPK by three complementary mechanisms: (1) promotion of phosphorylation of Thr172 in the α subunit kinase domain by the upstream kinase LKB1 (also known as STK11) (Hawley et al., 1996); (2) inhibition of Thr172 dephosphorylation by protein phosphatases (Davies et al., 1995); and (3) allosteric activation of AMPK already phosphorylated on Thr172 (Gowans et al., 2013; Yeh et al., 1980).

This canonical energy-sensing mechanism for AMPK activation can be triggered by agents that either inhibit glycolysis (e.g. 2-deoxyglucose) or mitochondrial ATP synthesis (e.g. oligomycin and metformin) (Hawley et al., 2010). However, such drugs can have many AMPK-independent off-target effects. The canonical mechanism can also be mimicked by pro-drugs that are converted by cellular metabolism into AMP mimetics. The original example was 5-aminoimidazole-4-carboxamide ribonucleoside (AICAR), which is converted into the AMP mimetic, ZMP. However, ZMP is ∼50-fold less potent than AMP (Corton et al., 1995), and AICAR has known off-target effects in cells and *in vivo* (Foretz et al., 2010; Longnus et al., 2003; Vincent et al., 1991). Much more specific is C13, a pro-drug that is converted into C2, an AMP analogue >100-fold more potent as an AMPK activator than AMP itself, although it only significantly activates complexes containing AMPK-α1 and not -α2 (Gomez-Galeno et al., 2010; Hunter et al., 2014).

AMPK is also activated by compounds that bind the allosteric drug and metabolite (ADaM) site (Langendorf and Kemp, 2015; Xiao et al., 2013). Most such compounds are synthetic molecules derived from screens that searched for allosteric activators of AMPK, such as A-769662 (Cool et al., 2006) and MK-8722 (Myers et al., 2017), although long chain fatty acyl-CoA esters are natural activators that bind the ADaM site (Pinkosky et al., 2020). A-769662 has known off-target AMPK-independent effects (Benziane et al., 2009; Foretz et al., 2010), but MK-8722 and other more recently developed activators are much more potent and therefore likely to be more specific.

The multiple isoforms of each AMPK subunit appear to have arisen during the two rounds of whole genome duplication that occurred during the evolution of vertebrates (Ross et al., 2016b). There are differences in their expression in different cell types (Cheung et al., 2000; Mahlapuu et al., 2004; Rolf et al., 2013; Stapleton et al., 1996; Thornton et al., 1998), and differences in the AMP- and ADP-mediated regulation of γ isoform function (Ross et al., 2016a). In addition, some activators that bind the ADaM site (e.g. A-769662 or long chain acyl-CoAs) only activate β1-containing complexes (Pinkosky et al., 2020; Scott et al., 2008). However, other distinct functions of the 12 heterotrimer combinations are currently unknown. One attractive idea addressed in the present study is that the variable regions in these isoforms target AMPK complexes to specific subcellular locations. Some evidence for this already exists – for example, some subunit isoforms (α1, α2, β2 and γ1) have been reported to localize at the outer mitochondrial membrane (OMM) (Drake et al., 2021). Moreover, both the β1 (Mitchelhill et al., 1997) and β2 (Oakhill et al., 2010) subunit isoforms are myristoylated at their N-termini, and this has been proposed to strengthen their association with membranes (Oakhill et al., 2010; Warden et al., 2001), although the membranes involved were not defined.

In the present study, we found that almost all AMPK isoform combinations are associated with the Golgi in different cell types. The Golgi is a central sorting station for different trafficking pathways; a key role is the processing and sorting of proteins destined for secretion, the plasma membrane, lysosomes or lipid droplets. One class of regulators of Golgi membrane trafficking are the ADP-ribosylation factors (ARFs), membrane-associated GTPases that recruit coat proteins or complexes required for membrane trafficking (Jackson, 2018). Some ARFs, including ARF1, are converted into their GTP-bound forms by the guanine nucleotide exchange factor (GEF) activity associated with the Sec7 domain of Golgi-specific Brefeldin A resistance factor-1 (GBF1), so-called because its overexpression confers resistance to the fungal toxin Brefeldin A (Claude et al., 1999). GBF1 function is also inhibited by the small molecule Golgicide A, which, unlike Brefeldin A, does not inhibit other Arf-GEFs such as the Brefeldin A-inhibited guanine nucleotide-exchange protein-1 and -2 (BIG1 and BIG2; also known as ARFGEF1 and ARFGEF2, respectively) (Saenz et al., 2009).

GBF1 is involved in retrograde trafficking from the Golgi cisternae back to the ER. Miyamoto et al. (2008) reported that AMPK phosphorylates GBF1 at Thr1337, and provided evidence that AMPK activation in intact cells causes the disassembly of the Golgi. Subsequent studies have suggested that phosphorylation of GBF1 by AMPK might be involved in the dispersion of the Golgi during mitosis (Mao et al., 2013). Activation of AMPK in endothelial cells also inhibited anterograde trafficking of von Willebrand factor from the Golgi to secretory granules, and inhibited its secretion in response to histamine (Lopes-da-Silva et al., 2019). However, in these studies activation or inhibition of AMPK utilized low glucose, 2-deoxyglucose, AICAR or compound C, all of which have off-target AMPK-independent effects as discussed above. Compound C in particular inhibits numerous other protein kinases more potently than AMPK (Bain et al., 2007). In this study, we show that AMPK associates with the Golgi in a manner that is independent of isoform composition and N-myristoylation of the β subunits. We also show using AMPK knockout and GBF1 mutant knock-in cells, as well as more specific AMPK agonists than those used previously, that phosphorylation of GBF1 at residue Thr1337 promotes Golgi disassembly and inhibits protein trafficking to the plasma membrane. Such effects are mechanistically similar to those seen upon treatment with the GBF1 inhibitor Golgicide A.

## RESULTS

### Targeting of AMPK isoform combinations to a juxtanuclear Golgi-like region in HeLa cells

**Fig. 1.**
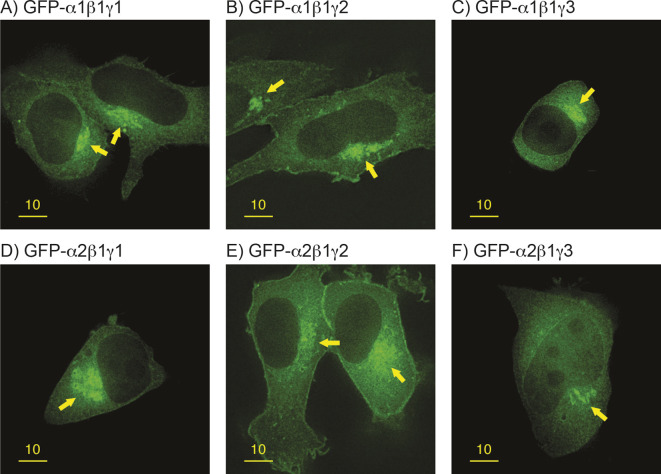
**Targeting of β1 complexes to different subcellular locations.** Images show fluorescence micrographs obtained by deconvolution microscopy (0.2 µm optical sections) of transfected HeLa cells co-expressing β1 with GFP–α1 (A–C) or GFP–α2 (D–F), and γ1 (A,D), γ2 (B,E) or γ3 (C,F). The enrichment of fluorescence adjacent to one pole of the nucleus is indicated by yellow arrows. Images representative of five repeats. Scale bars: 10 µm.

To study whether different combinations of subunit isoforms in AMPK heterotrimers might target them to different subcellular locations, we initially used transient transfection of tagged α subunits in human epithelial (HeLa) cells. We showed previously that both expression and activity of GFP-tagged α1 subunits after transient transfection were reduced >20-fold if plasmids encoding β and γ subunits were not co-transfected (Scott et al., 2002), whereas co-expression of β and γ subunits with either GFP- or Myc-tagged α subunits caused a modest increase (2- to 5-fold) in their expression and activity compared with endogenous levels (Hudson et al., 2003). The necessity to co-express β and γ subunits to obtain functional α subunit expression was useful because it allowed us to study the effect of specific combinations of β and γ isoforms on the targeting of GFP-tagged α subunits to specific subcellular locations. Fig. 1 shows GFP–AMPK-α [hereafter tagged AMPK subunits are denoted by subunit designation(s), e.g. GFP–α1] fluorescence for the six possible heterotrimeric combinations containing β1. Although there was a diffuse cytoplasmic fluorescence in all cases, we noted a clear and consistent enrichment of GFP–α subunit accumulation in a juxtanuclear region (Fig. 1 panels, arrowed), irrespective of which α or γ isoform was expressed. In addition, isoform combinations containing γ2 were targeted to the cell periphery, indicating accumulation at the plasma membrane (Fig. 1B,E).

We also co-expressed the six heterotrimeric combinations containing β2 rather than β1 (Fig. S1). The most striking feature in this case was the appearance of brightly fluorescent ovoid structures in the cytoplasm, which we have previously shown represent non-membrane bound inclusions in which AMPK aggregates with glycogen particles; these only occur when the expressed β subunits contain the carbohydrate-binding module (CBM) and are much more frequent in cells expressing β2 rather than β1 (Hudson et al., 2003), consistent with findings that the β2-CBM has a much higher affinity for glycogen (Koay et al., 2010). However, we also observed the enrichment of fluorescence in the juxtanuclear region (arrows), especially when co-expressing with GFP–α2 (Fig. S1D–F); it was less obvious using GFP–α1 (Fig. S1A–C).

### Targeting of γ2 complexes to the plasma membrane requires the N-terminal extension of γ2

One interesting finding was that upon co-expression of the γ2 isoform with β1 and GFP–α1 in HeLa cells, GFP fluorescence was enriched at the plasma membrane; such effects were not observed with γ1 or γ3. This was even more striking when such experiments were repeated in Chinese hamster ovary (CHO) cells (Fig. S2A–C). Given that the most unique feature of γ2 is its long, proline-rich N-terminal extension (NTE) of ∼250 amino acids, we co-expressed GFP–α1 and β1 with γ2 carrying a C-terminal FLAG tag, either using the full-length γ2 sequence (γ2L) or an N-terminally truncated version (244–569, γ2S). On co-expression of GFP–α1, β1 and γ2L–FLAG the GFP fluorescence and anti-FLAG signal (red) coincided at or near the plasma membrane, although there was still fluorescence in the juxtanuclear region (Fig. S2A–C). By contrast, when the truncated γ2S–FLAG variant was co-expressed with GFP–α1 and β1, the green and red fluorescence merged throughout the cytoplasm with the usual enrichment in the juxtanuclear region, but with no fluorescence evident at the cell periphery (Fig. S2D–F). This shows that the NTE of the γ2 subunit is required for targeting the AMPK complex to the plasma membrane.

To directly test the targeting role of the γ2-NTE in intracellular localization, we made constructs in which this region (residues 1–268, terminating just before the first CBS repeat of γ2), or C-terminal truncations of it, were fused at the N-terminus of YFP. When YFP alone was expressed in CHO cells, fluorescence was largely confined to the nucleus (Fig. S2G). By contrast, with the NTE–YFP fusions there was also clear fluorescence at the cell margin, at least with the 1–268, 1–191 and 1–159 constructs. With the 1–126 and 1–106 constructs this was much less obvious, and the fluorescence appeared to be dispersed throughout the cytoplasm and nucleus instead (Fig. S2H–L). Thus, the γ2-NTE targets YFP to the cell periphery and plasma membrane, and this requires the presence of residues between 126 and 159 of the γ2 isoform.

### The juxtanuclear region where AMPK is enriched represents the Golgi

**Fig. 2.**
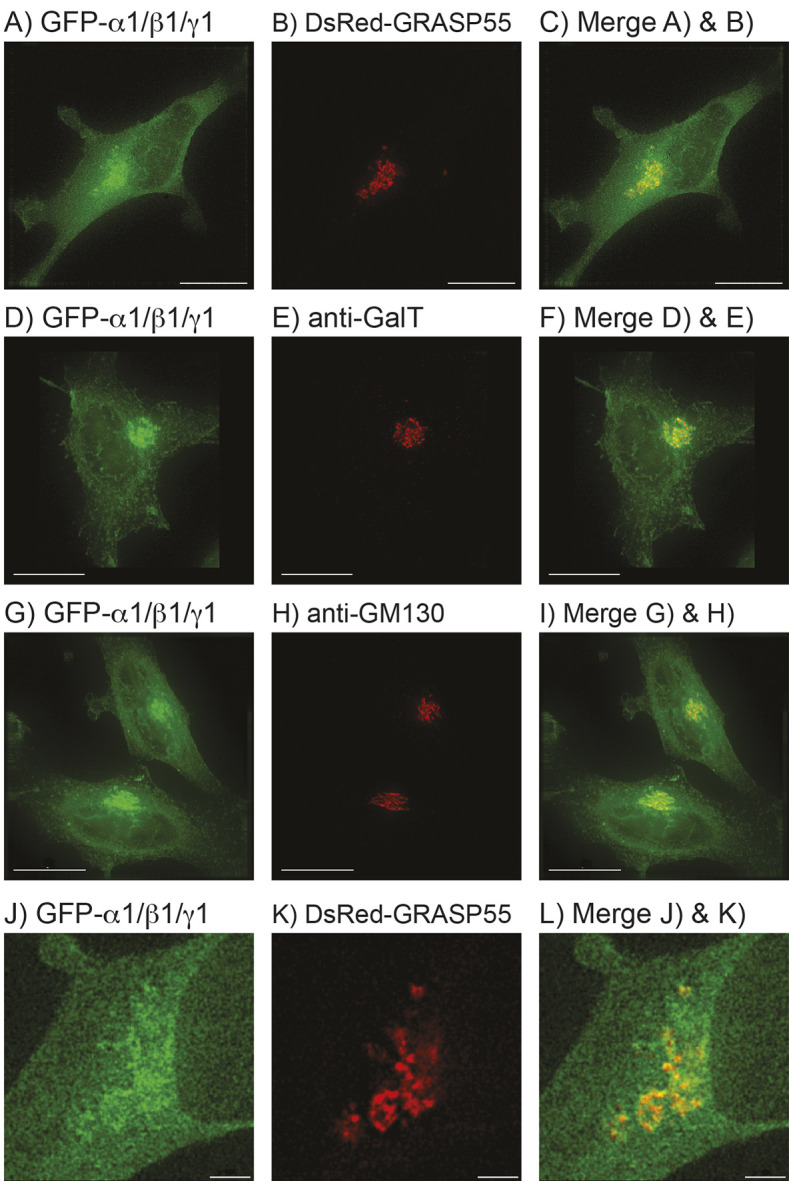
**The juxtanuclear region where AMPK is enriched contains the Golgi.** Images were obtained by deconvolution microscopy and are whole-cell projections of fixed HeLa cells co-transfected with GFP–α1, β1 and γ1, that had been either co-transfected with DNA encoding DsRed–GRASP55 (A–C,J–L), or counterstained with Texas Red-labelled antibodies against GalT (D–F) or GM130 (G–I). Left-hand panels show GFP fluorescence (green), centre panels show DsRed or Texas Red fluorescence, and right-hand panels show merged images. Images representative of at least three repeats. Scale bars: 15 µm (A–I); 2 µm (J–L).

We suspected that the juxtanuclear fluorescence evident in Fig. 1 and Figs S1 and S2A–SF represented the Golgi, and this was supported by expressing GFP–α1, β1 and γ1 and dual labelling with Golgi markers, either by co-transfection of a plasmid encoding a Discosoma Red protein–GRASP55 fusion (DsRed-GRASP55, a medial Golgi marker, Fig. 2A–C; GRASP55 is also known as GORASP2), or by immunofluorescence microscopy (IFM) using antibodies against β-1,4-galactosyl transferase (GalT, *trans*-Golgi marker, Fig. 2D–F) or GM130 (also known as GOLGA2; a *cis*-Golgi marker, Fig. 2G–I). Superimposition of the different staining patterns clearly showed overlap between AMPK subunits and Golgi markers (Fig. 2), although inspection at higher magnification, e.g. for DsRed–GRASP55 and GFP–α1β1γ1 (Fig. 2J–L), suggested that AMPK was present in a more extensive volume than any specific Golgi sub-compartment marker. This suggests that AMPK is enriched within the Golgi region but is not restricted to a specific sub-compartment.

### Enrichment of transfected AMPK at the Golgi does not require N-myristoylation of β subunits

Given that N-myristoylation often targets proteins to membranes [including ARF1 to the Golgi (Haun et al., 1993)], we wondered whether β subunit N-myristoylation might be involved in the Golgi enrichment of transfected AMPK complexes. We therefore constructed non-myristoylatable (G2A) mutants of β1–YFP or β2–YFP fusions and co-expressed with Myc–α1 and γ1 subunits. However, the enrichment of the YFP and anti-Myc signals in the juxtanuclear region was still observed, suggesting that N-myristoylation of β subunits is not essential for Golgi localization (Fig. S3). The merged images also suggest that the α and the β subunits of AMPK are associated with each other throughout the cell, including at the Golgi (Fig. S3).

### Endogenous AMPK is associated with the Golgi in U2OS cells

**Fig. 3.**
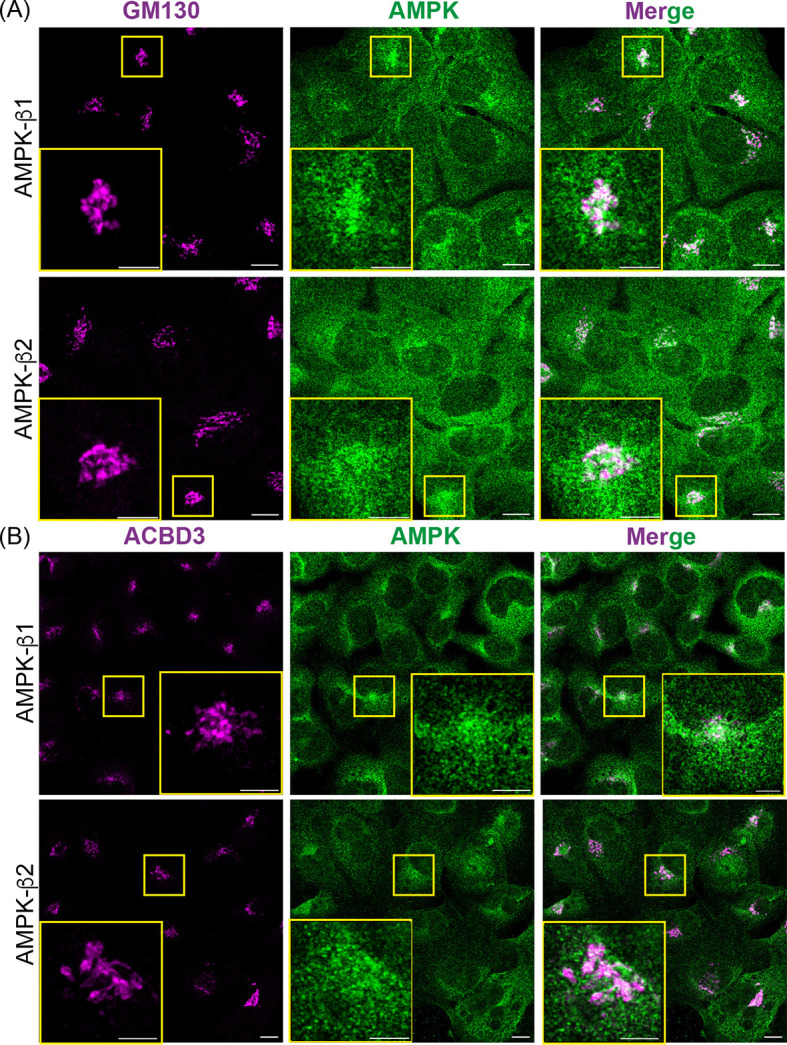
**Endogenous AMPK-β1 and -β2 are associated with the Golgi.** IFM (optical slices) of U2OS cells stained with anti-β1 or anti-β2 antibodies (green) reveals that they partially colocalize with (A) GM-130 (a *cis*-Golgi marker, magenta) or (B) ACBD3 (a medial-Golgi marker, magenta) as revealed by the merged images (right). The large yellow rectangles (insets) are the areas indicated by small yellow rectangles at higher magnification. Images representative of six repeats. Scale bars: 10 µm (main images); 5 µm (insets).

To study the subcellular localization of endogenous AMPK subunit isoforms, we switched to U2OS (human osteosarcoma) cells, whose shape and growth pattern make them ideal for microscope-based studies. We used U2OS cells carrying a single Flp recombinase target (FRT) site (Grumati et al., 2017), and created double knockouts (DKO) of the endogenous AMPK α1 and α2 (α1/α2) or β1 and β2 (β1/β2) subunits using the CRISPR:Cas9 (D10A nickase) method. Commercial antibodies against β1 and β2 were validated for use in IFM; immunoreactivity detected in the parental U2OS cells was lost in the β1/β2 DKO cells (Fig. S4). IFM on optical slices of U2OS cells stained with anti-β1 or -β2 antibodies showed that these subunits were spread throughout the cytoplasm and, at a lower level, within the nucleus. However, we also detected clusters of more intense staining within a juxtanuclear region that corresponded to the Golgi, as judged by colocalization with GM130 (a *cis*-Golgi marker) or ACBD3 (a medial-Golgi marker) (Fig. 3). The partial nature of Golgi colocalization of AMPK was reflected in relatively low Pearson's correlations of 0.17±0.02 or 0.16±0.01 (mean±s.d., *n*=6; β1 or β2 with GM130) and 0.16±0.01 or 0.16±0.02 (*n*=6, β1 or β2 with ACBD3).

### AMPK activation causes disaggregation of the Golgi via phosphorylation of Thr1337 on GBF1

**Fig. 4.**
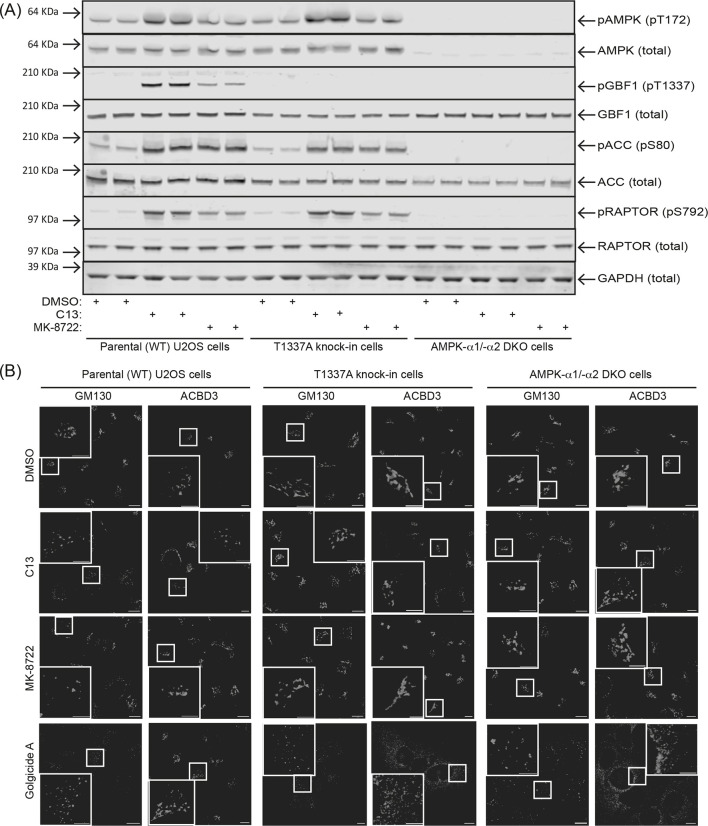
**Activation of AMPK and/or inhibition of GBF1 cause phosphorylation of AMPK targets including GBF1 and disaggregation of the Golgi.** (A) Duplicate dishes of parental (WT), T1337A knock-in or AMPK-α1/-α2 (α1/α2) DKO cells were treated for 1 h with 0.1% DMSO (vehicle control), 300 µM C13 or 200 nM MK-8722, cell lysates were analysed by SDS-PAGE and blots probed for the phosphorylated or total proteins shown. Image of blot representative of three repeats. (B) Parental (WT), T1337A knock-in or AMPK-α1/-α2 DKO cells were treated for 1 h with 0.1% DMSO, 300 µM C13, 200 nM MK-8722 or 1 µM Golgicide A, fixed, and stained with antibody against the *cis*-Golgi marker GM130. The large white rectangles (insets) are the areas indicated by smaller white rectangles at higher magnification. The results in B are quantified in Fig. S5. Scale bars: 10 µm (main images); 5 µm (insets).

Miyamoto et al. (2008) reported that AMPK activation causes GBF1 phosphorylation at Thr1337 and provided evidence that AMPK activation in intact cells causes disassembly of the Golgi, although they used rather non-specific AMPK activators (2-deoxyglucose and AICAR) and inhibitors (compound C). As a more rigorous test of AMPK-regulated GBF1 function, we used CRISPR/Cas9 gene editing in U2OS cells to generate a homozygous knock-in mutation in the *GBF1* locus expressing a non-phosphorylatable T1337A mutant protein. Treatment with the AMPK activators C13 (300 µM) or MK-8722 (200 nM) had similar effects on phosphorylation of acetyl-CoA carboxylase (ACC) at the primary AMPK site (Ser80), but MK-8722 had somewhat smaller effects on two other AMPK target phosphorylation sites (i.e. Thr1337 on GBF1 and Ser792 on RAPTOR) (Fig. 4A). MK-8722 also did not significantly increase Thr172 phosphorylation on AMPK-α itself, consistent with findings that ADaM site activators primarily work by allosteric activation rather than by promoting net Thr172 phosphorylation on AMPK-α (Foretz et al., 2010). In the GBF1-T1337A knock-in cell line, AMPK was activated normally as judged by Thr172 phosphorylation on AMPK-α as well as phosphorylation of ACC and RAPTOR, but detection of the GBF1-pT1337 epitope was, as expected, completely absent (Fig. 4A). Furthermore, expression of AMPK and phosphorylation of ACC, GBF1 and RAPTOR were all absent in α1/α2 DKO cells.

AMPK activation using C13 or MK-8722 caused a marked disaggregation of the Golgi apparatus, assessed using IFM with either *cis*-Golgi (GM130) or medial-Golgi (ACBD3) markers (Fig. 4B); these data were quantified for the *cis*-Golgi (Fig. S5). With the GM130 marker, the mean number of *cis*-Golgi elements increased 12-fold in response to C13 and 3- to 4-fold in response to MK-8722, consistent with the larger degree of GBF1 phosphorylation by the former (Fig. 4A); the effect of the GBF1 inhibitor Golgicide A was even greater (23-fold). The effects of C13 and MK-8722, but not Golgicide A, on this parameter were almost eliminated in both α1/α2 DKO and GBF1-T1337A cell lines. Similarly, there were large decreases in the mean size of Golgi elements and very large increases in the proportion of cells with fragmented Golgi that, in the case of effects of C13 and MK-8722 but not Golgicide A, were reduced or eliminated in the DKO and T1337A cells; by contrast, the mean area of Golgi vesicles per cell was unchanged by any of the three treatments (Fig. S5).

### Effects of AMPK activators and Golgicide A on colocalization of GBF1 and AMPK with the Golgi

**Fig. 5.**
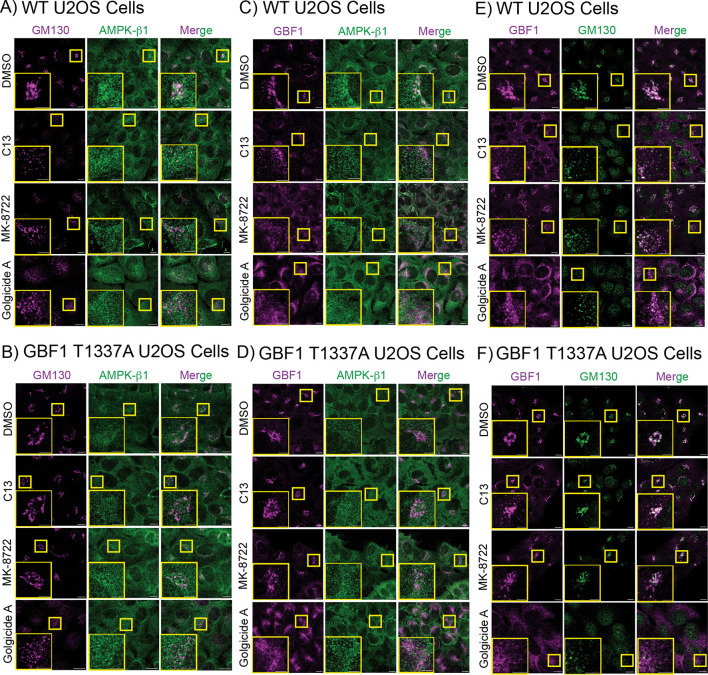
**Changes in colocalization of GM130 and GBF1 with AMPK-β1, and GBF1 with GM130 in WT and T1337A knock-in cells treated with C13, MK-8722 or Golgicide A.** Cells were incubated with 0.1% DMSO, 300 µM C13, 200 nM MK-8722 or 1 µM Golgicide A for 1 h. The images were obtained by staining with the indicated antibodies in the left-hand and centre panels, with merged images shown in the right-hand panels. Quantification (changes in Pearson's correlation) are shown in Fig. S6. The small yellow rectangles show the areas that are enlarged in the inset (large yellow rectangles). Scale bars: 10 µm (main images); 5 µm (insets).

The effects of C13, MK-8722 or Golgicide A on colocalization of GM130 and AMPK-β1, GBF1 and AMPK-β1, or GBF1 and GM130, are shown in Fig. 5; the data were quantified as changes in Pearson's correlations (Fig. S6). In control (DMSO-treated) cells, GM130 and GBF1 showed a relatively high mean correlation of ≈0.5, but this declined markedly in cells treated with C13, MK-8722 or Golgicide A, suggesting that phosphorylation of GBF1 or Golgicide A treatment causes it to dissociate from the *cis*-Golgi. As already pointed out, GM130 and AMPK-β1 showed a relatively low mean correlation (≈0.18) in control cells, but this correlation declined still further in cells treated with C13, MK-8722 or Golgicide A, suggesting that AMPK also dissociates from the *cis*-Golgi. At the same time, the relatively low correlation between AMPK-β1 and GBF1 (0.18) also declined significantly in cells treated with C13, MK-8722 or Golgicide A. Interestingly, all of the effects of C13 and MK-8722, but not Golgicide A, were abolished in GBF1-T1337A cells (Fig. S6).

### Association between AMPK, GBF1 and the Golgi assessed by purification of Golgi vesicles

**Fig. 6.**
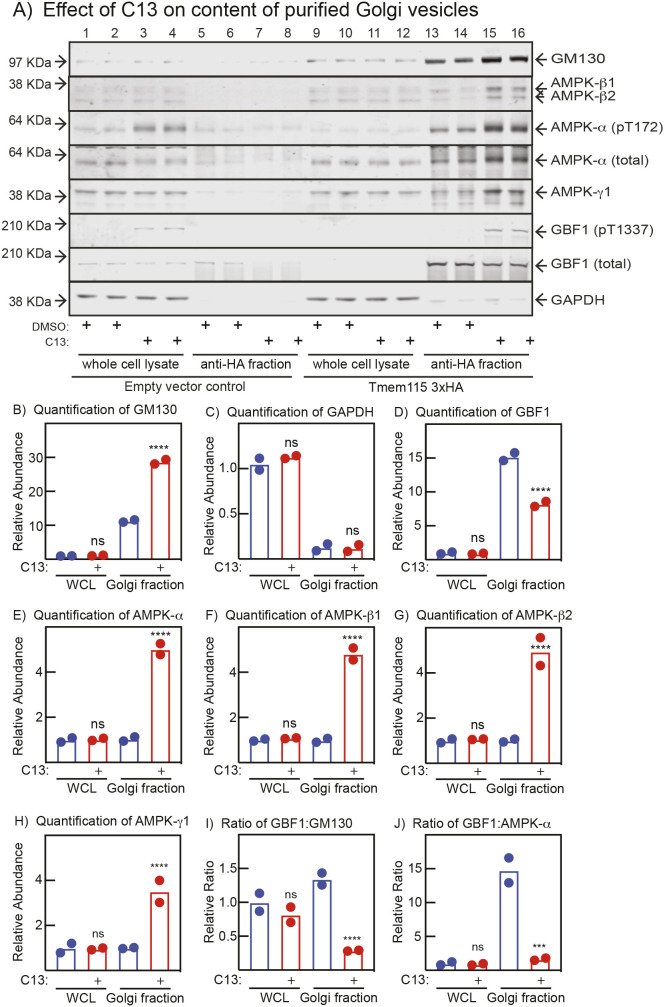
**Purification of Golgi vesicles from lysates of WT U2OS cells expressing a triple HA-tagged version of the Golgi membrane protein Tmem115 and treated with or without C13.** (A) Western blots, (B) quantification of western blots. (A) Lanes 1–4 and 5–8 are from cells that had been mock-transfected with empty vector and lanes 9–12 and 13–16 are from cells expressing triple HA-tagged Tmem115. Cells were treated with 0.1% DMSO (control) or 300 µM C13 for 1 h. Samples loaded were either whole-cell lysates (1–4, 9–12) or fractions binding to anti-HA antibodies coupled to magnetic beads (5–8, 13–16). Sample equivalent to 40 µg of whole-cell lysate protein was loaded in each lane. (B) Quantification by densitometry of the western blots in A; results are mean and actual data points (*n*=2), and only results for the cells expressing triple HA-tagged Tmem115 are shown. Asterisks show mean values significantly different from controls without C13: *P<0.05, **P<0.01, ***P<0.001, ****P<0.0001; ns, not significant (one-way ANOVA with Holm–Sidak post test).

We also analysed the association between subunits of the AMPK complex, GBF1 and the Golgi by generating U2OS cells stably expressing a triple haemagglutinin (3×HA)-tagged Tmem115 using viral transduction (Abu-Remaileh et al., 2017). Tmem115 is a transmembrane protein present in all Golgi sub-compartments (Ong et al., 2014). Golgi vesicles were affinity purified from these cells using magnetic beads linked to anti-HA antibodies. Purified 3×HA–Tmem15 vesicles were relatively pure as judged by a 10-fold enrichment compared with the whole-cell lysate (WCL) of the *cis*-Golgi marker GM130 (Fig. 6A,B) and a 5- to 10-fold depletion of the cytoplasmic marker glyceraldehyde phosphate dehydrogenase (GAPDH) (Fig. 6A,C). Interestingly, treatment with the AMPK activator C13 increased the enrichment of GM130 from 10- to 30-fold. In control (DMSO-treated) cells, GBF1 was enriched 15-fold in the Golgi vesicles compared with the WCL (Fig. 6A,D), but this decreased by almost 50% on treatment with C13, which was also evident as a large decrease in the GBF1:GM130 ratio (Fig. 6I). This was accompanied by a phosphorylation of Thr1337 on GBF1 that was evident in the Golgi fraction (Fig. 6A). In control (DMSO-treated) cells the α (total), β1, β2 and γ1 subunits of AMPK were all present in the Golgi fraction at about the same abundance as in the WCL, but their levels in the former increased 4- to 5-fold on treatment with C13 (Fig. 6E–H), accompanied by an increased phosphorylation of Thr172 on AMPK-α (Fig. 6A). Finally, the GBF1:AMPK-α ratio decreased markedly after C13 treatment, consistent with the idea that GBF1 dissociates from the Golgi upon Thr1337 phosphorylation by AMPK.

### AMPK activation still causes Golgi fragmentation in cells expressing non-myristoylatable β1 or β2 subunits

**Fig. 7.**
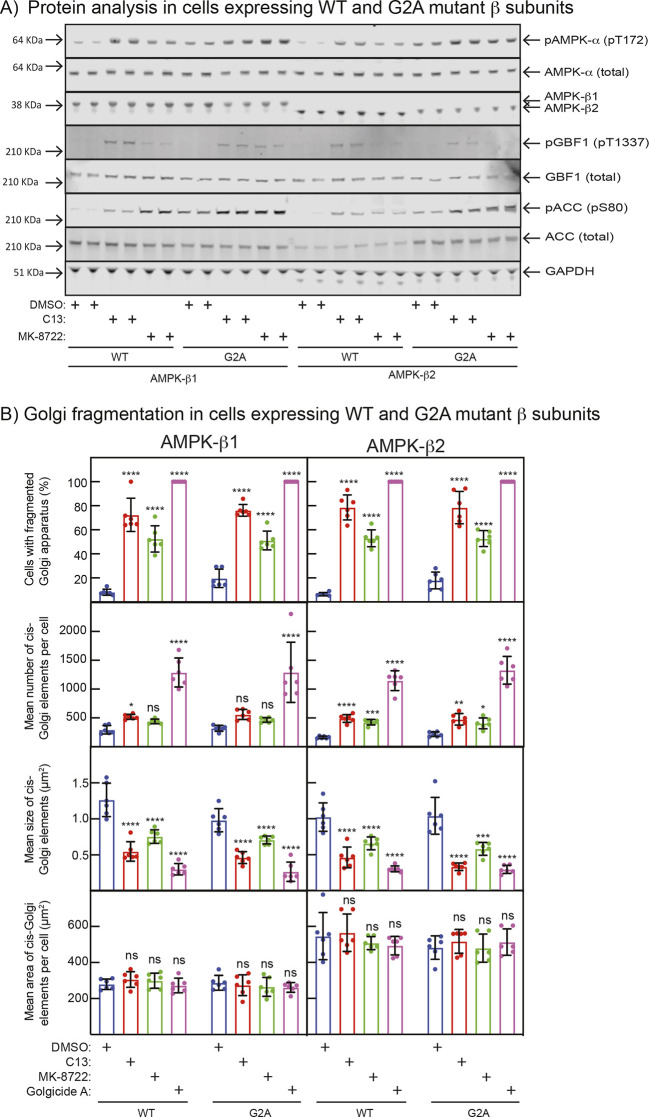
**Fragmentation of the Golgi induced by AMPK activation is not dependent on the myristoylation status of AMPK-β1 or -β2.** (A) Cells stably expressing WT or non-myristoylatable (G2A) mutants of AMPK-β1 or -β2 were treated for 1 h with 0.1% DMSO (control), 300 µM C13 or 200 nM MK-8722, lysates prepared and analysed by western blotting with the antibodies shown. The anti-AMPK-β antibody used recognizes both β1 and β2, but they can be distinguished by differing mobilities on SDS-PAGE. (B) Cells expressing WT or G2A mutants were treated for 1 h with 0.1% DMSO, 300 µM C13, 200 nM MK-8722 or 1 µM Golgicide A, IFM images prepared using anti-GM130 antibodies and quantified as in Fig. S5. Results are mean±s.d. for six biological replicates in each case. Mean values significantly different from DMSO controls are indicated: *P<0.05, **P<0.01, ***P<0.001, ****P<0.0001; ns, not significant (one-way ANOVA with Holm–Sidak post test).

To further investigate potential roles of N-myristoylation of the AMPK-β subunits on the functions of AMPK at the Golgi apparatus, we used AMPK-β1/β2 DKO cells and stably re-expressed via their Flp recombinase target site either β1 or β2, in both cases as either wild type (WT) or non-myristoylatable (G2A) mutants. Western blotting data (Fig. 7) showed that the WT and G2A mutants of β1 and β2 were expressed at similar levels as judged by probing blots using a pan-β antibody. Phosphorylation of AMPK itself, or of GBF1 and ACC, in response to C13 or MK-8722 were also similar in all four cell lines except that the basal Thr172 phosphorylation and activity of AMPK activity appeared to be higher with the G2A mutants (both for β1 and β2) as judged by increased phosphorylation of AMPK, GBF1 and ACC.

Fig. S7 shows fragmentation of the Golgi in these cells, as assessed by IFM using staining with anti-GM130 that is quantified in Fig. 7B. The results were very similar to those for the parental WT cells in Fig. 4 and Fig. S5. Remarkably similar findings were observed between the WT and G2A mutants of β1 or β2, except that the G2A mutants displayed a larger proportion of cells with fragmented Golgi in the DMSO controls, consistent with the higher basal activity of AMPK evident in Fig. 7A.

### AMPK activation inhibits the constitutive secretory pathway via phosphorylation of GBF1

**Fig. 8.**
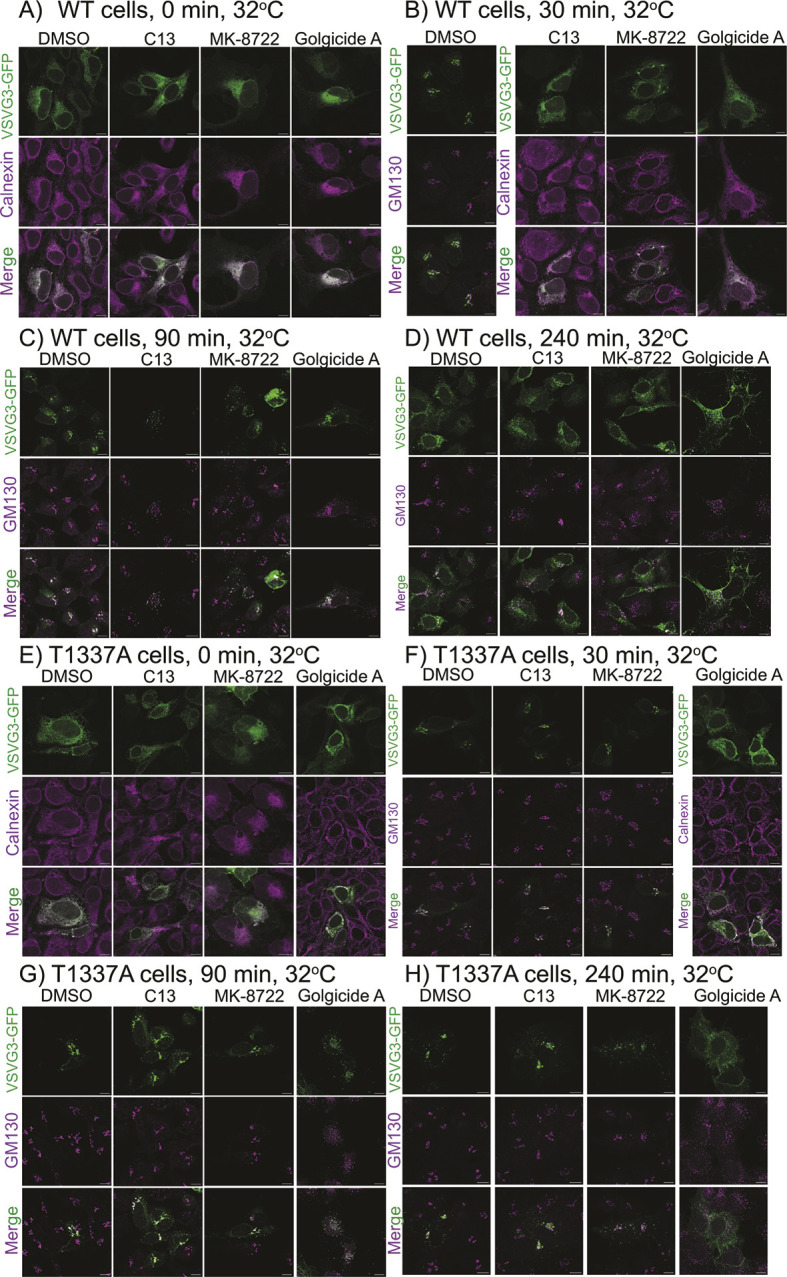
**Transit of temperature-sensitive VSVG3–GFP from the ER to the plasma membrane via the Golgi is inhibited by AMPK activation or GBF1 inhibition.** U2OS cells were transfected with DNA encoding VSVG–GFP for 6 h at the restrictive temperature (40°C), which causes the protein to misfold and accumulate in the ER. Cells were then treated with 0.1% DMSO (control), 300 µM C13, 200 nM MK-8722 or 1 µM Golgicide A. After 1 h (defined as time zero) cells were transferred to the permissive temperature (32°C). At the indicated times cells were fixed and stained with antibodies against markers for the ER (calnexin) or Golgi (GM130) and *Z* sections (1 µM) were visualized in the confocal microscope. Results for six biological replicates are quantified in Fig. S8. Scale bars: 10 µm.

A major function of the Golgi is the sorting and processing (e.g. glycosylation) of proteins destined for distal compartments such as plasma membranes, endosomes and lysosomes, or for secretion. This is an important biosynthetic activity in most cells, and both membrane trafficking and protein processing would be expected to consume large quantities of cellular ATP. We therefore suspected that AMPK activation, which is triggered by ATP depletion, would inhibit these processes as an energy-conserving mechanism. To test this, we transfected into parental (WT) and GBF1-T1337A knock-in U2OS cells a GFP-tagged temperature-sensitive mutant of the viral membrane protein VSVG (Toomre et al., 1999). At the restrictive temperature (40°C) the VSVG–GFP protein is misfolded and retained in the ER, but on transfer to the permissive temperature (32°C), VSVG folds correctly and transits from the ER to the Golgi and eventually to the plasma membrane. We analysed VSVG–GFP transfected parental (WT) or GBF1-T1337A knock-in cells that had been treated with AMPK activators or Golgicide A for 1 h at the restrictive temperature (40°C) and then transferred to the permissive temperature. Samples were analysed at the time of transfer to 32°C and at timed intervals thereafter; images from one experiment are shown in Fig. 8 and quantification of several experiments in Fig. S8. In controls (DMSO-treated) the VSVG–GFP protein had largely left the ER by 30 min and had reached a peak in the Golgi, then declining slowly in the Golgi as it moved to the plasma membrane. We also attempted to quantify colocalization of VSVG–GFP and a plasma membrane marker (Na^+^/K^+^ ATPase). However, the results were difficult to interpret mainly because of the flattened growth form of U2OS cells, which meant that rather diffuse areas of any *Z*-section stained positive for the marker, making it difficult to optically section plasma membrane and cytosol using our microscopy technique.

When the WT cells were treated with the AMPK activators C13 or MK-8722 for 1 h prior to transfer to the permissive temperature, there was a marked delay in the transfer of VSVG–GFP to the Golgi and beyond, such that its appearance in the Golgi now peaked at 90 min, rather than 30 min as in the controls; very similar results were obtained with Golgicide A. We also assessed VSVG–GFP secretion in GBF1-T1337A mutant knock-in cells (Fig. 8E–H; Fig. S8B). Here, results in cells treated with C13 or MK-8722 were indistinguishable from those with DMSO controls, although the effects of Golgicide A remained. Overall, these findings confirm that the fragmentation of the Golgi produced by these agents interferes with the trafficking of a membrane protein through the Golgi towards the plasma membrane, and in the case of AMPK activators that this is mediated by the phosphorylation of GBF1 at Thr1337, an effect that can be mimicked using the GBF1 inhibitor, Golgicide A.

## DISCUSSION

The major findings that emerge from this study are that AMPK is associated with the Golgi in the basal state, and that its activation leads to Golgi fragmentation and inhibition of protein trafficking via the Golgi. These effects are mediated by AMPK-dependent phosphorylation of the guanine nucleotide exchange factor GBF1 on residue Thr1337. Our study has demonstrated AMPK association with the Golgi by three independent approaches: (1) expression of GFP-, YFP- or Myc-tagged α1, α2, β1 or β2 subunits with the other subunits of the complex, with demonstration of colocalization with Golgi markers; (2) colocalization with Golgi markers of endogenous β1 or β2 subunits by IFM, using antibodies validated in knockout cells; and (3) affinity purification of Golgi vesicles from cells expressing a HA-tagged Tmem115, which demonstrated the presence of AMPK-α, -β1, -β2 and -γ1 subunits in the Golgi fraction.

What is the function of AMPK at the Golgi? Miyamoto et al. (2008) provided evidence that AMPK phosphorylates the guanine nucleotide exchange factor GBF1 at Thr1337, and that AMPK activation in intact cells causes a fragmentation of the Golgi similar to that caused by Brefeldin A, a GBF1 inhibitor. Although this was certainly a prescient study, the agonists used to activate AMPK (2-deoxyglucose and AICAR) are now known to be relatively non-specific. For example, 2-deoxyglucose inhibits glycolysis and thus depletes cellular ATP (Hawley et al., 2010), whereas AICAR has many off-target effects (Foretz et al., 2010; Longnus et al., 2003; Vincent et al., 1991). To inhibit AMPK compound C was used, but this drug inhibits many other protein kinases and is not recommended as an AMPK inhibitor (Bain et al., 2007). Miyamoto et al. did also utilize expression of a dominant-negative mutant, but this approach can also have off-target effects. Our study now confirms and extends their original observations using much more specific agonists and genetic tests. To activate AMPK we used C13 and MK-8722, which act by different mechanisms – C13 is converted into the AMP mimetic C2 by cellular metabolism (Gomez-Galeno et al., 2010), whereas MK-8722 binds to the ADaM site (Myers et al., 2017). These distinct mechanisms mean that it is unlikely that the two agents would share common off-target effects. Moreover, we have shown that the effects of C13 or MK-8722 on phosphorylation of AMPK targets (GBF1, ACC and RAPTOR) all disappeared in α1/α2 DKO cells, whereas the phosphorylation of GBF1 at Thr1337 disappeared in a GBF-T1337A knock-in mutant cell line made by gene editing. Importantly, the Golgi fragmentation induced by C13 or MK-8722 was almost completely eliminated in both the α1/α2 DKO and the T1337A cells. As GBF1 has been reported to be phosphorylated at no less than 86 serine, threonine or tyrosine residues (Walton et al., 2020), it is remarkable that the loss of just one of these sites due to the T1337A mutation almost completely blocks the effects of AMPK activation on Golgi structure and function. Thr1337 lies in a large linker region of GBF1 between two domains termed homology downstream of Sec7 domain 2 and 3 (HDS2 and HDS3); this linker region contains numerous phosphorylation sites (Walton et al., 2020). Miyamoto et al. (2008) showed that AMPK phosphorylated GBF1 at Thr1337 in cell-free assays, suggesting that this is a direct phosphorylation. Certainly, the sequence around Thr1337 in GBF1 is an excellent fit for the consensus recognition motif for AMPK, with basic residues at the −3, −4 and −6 positions, and hydrophobic residues at the −5 and +4 positions, all of which are positive determinants (Steinberg and Hardie, 2023). This sequence motif is also conserved in vertebrates from humans to zebrafish (Miyamoto et al., 2008), suggesting that this mechanism is potentially relevant throughout vertebrates. Interestingly, Thr1337 on GBF1 is one of the relatively few sites modified on threonine (rather than serine) that are now well-established cellular targets for AMPK (Steinberg and Hardie, 2023). Although neither Miyamoto et al. (2008) nor ourselves addressed whether phosphorylation of Thr1337 directly affects the ARF1-targeted guanine nucleotide exchange activity of GBF1, Thr1337 phosphorylation appears to inhibit GBF1 function, thus blocking Golgi membrane traffic. Notably, pharmacological activators of AMPK show similar effects to those seen upon treatment with the GBF1 inhibitor Golgicide A, although the effects of Golgicide A (unlike those of C13 or MK-8722) were not abolished in the α1/α2 DKO or GBF1-T1337A cells.

The colocalization of the *cis*-Golgi marker GM130 and GBF1 decreased markedly after C13 treatment, with the Pearson's correlation dropping from ≈0.5 to ≈0.1, with smaller effects seen for MK-8722 and Golgicide A. The effects of C13 and MK-8722, but not Golgicide A, were abolished in the GBF1-T1337A knock-in cells. These results indicate that GBF1 dissociates from the *cis*-Golgi stacks upon phosphorylation of Thr1337, or upon Golgicide A inhibition of GBF1, and it is possible that this contributes to the overall inhibition of GBF1 function. Similar but smaller effects on the colocalization of GM130 and AMPK-β1, and of AMPK-β1 and GBF1 (albeit starting at much lower Pearson's correlation of 0.15–0.20) were also seen in response to all three agents, with the effects of C13 and MK-8722, but not Golgicide A, once again being abolished in the GBF1-T1337A knock-in cells. Our findings suggest that AMPK dissociates from GBF1 and from the *cis-*Golgi following phosphorylation of GBF1 on Thr1337.

Purification of Golgi vesicles from cells expressing a triple HA-tagged Golgi membrane protein (Tmem115) revealed a 10-fold enrichment in the Golgi marker GM130 and a depletion of 80–90% in the cytoplasmic marker GAPDH. Interestingly, all three AMPK subunits (α1/α2 detected using a pan-α antibody, β1 and β2, and γ1) were also present within the purified Golgi vesicles containing Tmem115 at a similar abundance to in the WCL. After treatment of cells with C13, the enrichment of GM130 increased to 30-fold. The reason for this is not clear, although one possibility is that the accompanying fragmentation of the Golgi stacks allows an increased interaction of the tagged Tmem115 protein with the anti-HA antibodies. After C13 treatment, the abundance of GBF1 in the Golgi vesicles decreased by half, or by ∼80% expressed as a ratio with GM130. This agrees with our microscopy studies suggesting that GBF1 dissociates from the cis-Golgi after C13 treatment, in a manner dependent upon phosphorylation of Thr1337 on GBF1. By contrast, the abundance of all AMPK subunits in the Golgi vesicles appeared to increase after C13 treatment, although this was partially counteracted by the even greater increased abundance of GM130 in this fraction. These biochemical data appear to contradict our microscopy studies, in which the colocalization of GM130 and AMPK-β1 decreased modestly upon treatment with C13 or MK-8722. One possibility is that AMPK partly redistributes to different Golgi membranes that express Tmem115 but not GM130. In any case, one firm conclusion using these different approaches is that AMPK and GBF1 redistribute to different locations following AMPK activation and Thr1337 phosphorylation on GBF1.

One question that has not been resolved by this study is the role of N-myristoylation of the AMPK-β1 and -β2 subunits, although we have shown that this modification is not required for the partial localization of the α or β subunits at the Golgi, nor for the effects of C13 or MK-8722 on Golgi structure. The one consistent difference that we noticed between the myristoylatable WT and non-myristoylatable G2A mutants of AMPK-β1 or -β2 is that the latter have increased basal activity, as evidenced by increased phosphorylation of Thr172 on AMPK-α and Ser80 on ACC, and an increased proportion of cells with fragmented Golgi under basal conditions when expressing the G2A mutants. This is consistent with previous studies of G2A mutants in different contexts (Neopane et al., 2022; Oakhill et al., 2010).

What is the physiological significance of Golgi fragmentation and disassembly by AMPK-mediated phosphorylation of Thr1337 on GBF1? This effect was accompanied by disruption of trafficking of a temperature-sensitive GFP-tagged plasma membrane protein (VSVG–GFP) from the ER to secretory vesicles via the Golgi. Cells treated with AMPK activators or Golgicide A displayed up to a 60 min delay in VSVG–GFP trafficking. Such delaying effects of C13 or MK-8722, but not Golgicide A, were abolished in GBF1-T1337A knock-in cells, confirming that the AMPK activators were acting by phosphorylation of GBF1 at that site. Given that membrane trafficking and covalent modification (e.g. glycosylation) of proteins passing through the Golgi must consume large amounts of ATP, this can be viewed as an important energy-conserving measure initiated by AMPK, which is a sensor of cellular energy status that is activated by ATP depletion.

One secondary finding of our study was that co-expression of AMPK-γ2, but not -γ1 or -γ3, with β1 and GFP–α1 in HeLa cells led to an increased distribution at the cell periphery, either at or close to the plasma membrane. An identical distribution was found for a C-terminally FLAG-tagged γ2 subunit when co-expressed with GFP–α1 and β1 in CHO cells from the Chinese hamster, indicating that the effect might have been conserved during evolution. Although we have not established the precise molecular basis for this phenomenon, we have found that it requires the long proline-rich N-terminal extension (NTE) of ∼250 residues in γ2 that is not found in γ1 or γ3. Removal of the NTE (residues 1–243) abolishes the GFP fluorescence at or near the membrane, redistributing it into the cytoplasm and the Golgi instead. This truncation rather precisely removes the NTE that is not present in γ1 or γ3, and the encoded protein is just one residue shorter than a γ2 variant (PRKAG2-b) that might result from use of an alternative transcriptional start site (Lang et al., 2000). As an alternative approach, we constructed fusions of the γ2-NTE (residues 1–268), and various C-terminal truncations, to YFP to show that a full-length γ2-NTE, in the absence of the remainder of γ2, could target YFP to a similar location at the cell margin; this required the γ2 sequence between residues 126 and 159.

Overall, our findings support our original hypothesis that different combinations of subunit isoforms target AMPK heterotrimers to different subcellular localizations given that the N-terminal extension of AMPK-γ2 targeted AMPK to the cell margin or plasma membrane. However, the major finding of this study, that AMPK is enriched at the Golgi, appeared to be independent of the subunit isoform composition, as all 12 isoform combinations were enriched at a juxtanuclear location that was shown to be the Golgi. Although we have not identified the detailed mechanism by which AMPK is enriched at the Golgi, we have shown that activation of AMPK causes phosphorylation of Thr1337 on GBF1 and the consequent disaggregation of the Golgi. Although this mechanism was first proposed more than a decade ago (Miyamoto et al., 2008), at that time the methods used to activate (2-deoxyglucose or AICAR) and inhibit (compound C) AMPK are now known to be relatively non-specific. We have been able to confirm the mechanism using two more potent and specific activators (C13 and MK-8722) that work by different mechanisms. Moreover, using α1/α2 double knockout and GBF1-T1337A knock-in cells, we have been able to prove that the effects of C13 and MK-8722 on Golgi disaggregation are dependent not only on the presence of AMPK but also on its phosphorylation of Thr1337. Finally, the major function of the Golgi is the post-translational modification (e.g. glycosylation) of secreted and membrane proteins and their sorting to specific cellular membranes. In this study, we have shown that trafficking of a temperature-sensitive mutant of VSVG, a viral protein that is glycosylated and constitutively trafficked to the plasma membrane at the permissive temperature, was inhibited by AMPK activation. Given that glycosylation and membrane trafficking consume large quantities of cellular ATP, we view this as yet another energy conservation mechanism for the AMPK system.

## MATERIALS AND METHODS

### Chemicals

Benzamidine, DMSO, dithiothreitol (DTT), HEPES, MgCl_2_, PMSF, Serva Blau G, Soya bean trypsin inhibitor (SBTI), *tris*(hydroxymethyl)aminomethane (Tris), Triton X-100 and Tween-20 were from Merck, Sharp and Dohme (Hertfordshire, UK). Sodium azide, EGTA, EDTA, NaCl, NaF and Na pyrophosphate were from BDH (Lutterworth, UK). Ethanol, methanol and orthophosphoric acid were from VWR (Lutterworth, UK). AMP, ATP and complete EDTA-free protease cocktail inhibitor were from Roche (Lewisham, UK). Glutathione–Sepharose beads and Optisafe HiSafe II liquid scintillation fluid were from Perkin-Elmer (Buckinghamshire, UK). Gibco Dulbecco's modified Eagle's medium (DMEM) GlutaMax™ high glucose, DMEM, Opti-MEM™ reduced serum medium, fetal bovine serum (FBS), trypsin-EDTA, sodium pyruvate and dialysed FBS were from Life Technologies (Renfrewshire, UK).

### DNA manipulation

The restriction enzymes Kpn1, Xho1, Bamh1 and Not1, and 3.1 ligation buffer, 10× reaction buffer, and 1 kb DNA ladder were from NEB (Hertfordshire, UK). DNA loading dye (6×), Top10 competent *E. coli* cells, XL Gold super-competent *E. coli* cells, molecular biology grade agarose, Lipofectamine™ 3000 and SOC medium were from Life Technologies (Renfrewshire, UK). T4 DNA ligase and FuGENE® 6 Transfection Reagent (FRIDGE) from Promega (Southampton, UK). QIA prep Spin Miniprep, QIA Quick Gel Extraction, HiSpeed Plasma Maxi, and QIA RNA Extraction kits were obtained from QIAGEN (Manchester, UK). LB medium, agar plates supplemented with 100 μg/ml ampicillin, sterile deionized water, PBS (10×), and TAE buffer (50×; 1× contains 40 mM Tris-acetate pH 8.0, 1 mM EDTA) were from Media Services, School of Life Sciences, University of Dundee, UK. Cloning was performed using the Strata Clone Blunt PCR Cloning Technology and PCR was performed using a Bio-Rad iCycler. One shot TOP10 (Aligent) chemically competent *E. coli* cells were used for transformation and the plasmid DNA was isolated using either QIAGEN^®^ HiSpeed^®^ Plasmid Mini-prep or Maxi-prep kits. DNA was purified using 1.5% agarose gel and extracted using the QIAGEN^®^ QIAquick^®^ Gel Extraction kit. DNA sequencing was performed by the MRC PPU Reagents and Services Sequencing Service, School of Life Sciences, University of Dundee, UK, using Applied Biosystems Big-Dye Version 3.1 on an Applied Biosystems model 3730 automated capillary DNA sequencer. Analysis was performed using DNADynamo version 1.0 (BlueTractorSoftware Ltd).

### Plasmids

pcDNA3-based plasmids encoding AMPK subunits (Cheung et al., 2000; Thornton et al., 1998; Woods et al., 1996b) were gifts from David Carling (Imperial College London, UK). Plasmids encoding N-terminally GFP-tagged α1 and α2 subunits were constructed in the expression vector phGFP-S65T (Salt et al., 1998). A plasmid encoding a GFP-tagged version of the VSV3 protein (VSVG3–GFP) (Toomre et al., 1999) was a gift from Kai Simons (Max Planck Institute, Dresden, Germany). A plasmid encoding a DsRed-tagged version of the Golgi protein GRASP55 (Baillie et al., 2002; Shorter et al., 1999) was a gift from Miles Houslay (University of Glasgow, UK). pcDNA5 FRT TO plasmids encoding AMPK-β1 or β2 (WT or G2A mutant) were provided by Dr Fiona Ross, University of Dundee, UK. pBabeD:hygro plasmid encoding Tmem115 3×HA (cat no. DU67960) was obtained from MRC PPU Reagents and Services, University of Dundee, UK.

### Antibodies

Monoclonal mouse anti-galactosyltransferase (GalT) was a gift from Tatsuo Suganuma (Miyazaki, Japan). The following primary antibodies and other probes were from commercial sources: mouse monoclonal anti-AMPK-pan-α (ab80039; RRID:AB_1603618), rabbit monoclonal anti-AMPK-β1 (ab32112; RRID:AB_722767), rabbit polyclonal anti-AMPK-β2 (ab135632, RRID:AB_2893182), rabbit polyclonal anti-calnexin (ab22595; RRID:AB_2069006), rabbit monoclonal anti-GM130 (ab52649) and rabbit monoclonal anti-TMEM192 (ab185545; RRID:AB_3095683) from Abcam; mouse monoclonal anti-ACBD3 (WH0064746M1; RRID:AB_2220068) and mouse monoclonal anti-GAPDH (MAB374; RRID:AB_2107445) from Merck; and phosphospecific (pT172) rabbit polyclonal anti-AMPK-α (2535L; RRID:AB_331250), rabbit monoclonal anti-AMPK-pan-β (4150; RRID:AB_10828832), mouse monoclonal anti-HA (2367; RRID:AB_10691311), phosphospecific (pS792) rabbit monoclonal anti-RAPTOR (89146; RRID:AB_2934061), rabbit monoclonal anti-RAPTOR (2280S; RRID:AB_561245; RRID:AB_331783) and mouse monoclonal anti-Myc (2276; RRID:AB_331783) from Cell Signaling Technology. Mouse monoclonal anti-GAPDH (MAB374; RRID:AB_2107445) and peroxidase-conjugated ExtrAvidin (E2886; RRID:AB_2620165) were from Sigma-Aldrich. The following secondary antibodies or probes were from commercial sources: FITC-conjugated anti-sheep-IgG, and Texas Red-conjugated anti-mouse-IgG and anti-rabbit-IgG secondary antibodies were from Jackson Laboratories. Alexa Fluor® 488-labelled donkey anti-rabbit-IgG (A11034; RRID:AB_2576217), Alexa Fluor® 594-labelled donkey anti-rabbit-IgG (A21207; RRID:AB_141637), Alexa Fluor® 488-labelled goat anti-mouse-IgG (A11029; RRID:AB_2534088), Alexa Fluor® 594-labelled goat anti-mouse-IgG (A11032; RRID:AB_2534088) and Streptavidin DyLight 800 (21851) were from Thermo Fisher Scientific. IRDye 700 and IRDye 800 were from LI-COR^®^ Biotechnology. Concentrations or dilutions of antibodies used are listed in Tables S1 and S2.

### Cell culture and transfection

HeLa cells were from the European Collection of Cell Cultures, and were validated by STR profiling (Public Health England, certificate dated 08/14/2015). U2OS cells bearing a Flp recombinase target site (Grumati et al., 2017) were a gift from David McEwan, Cancer Research UK Beatson Institute, Glasgow, UK. HeLa and U2OS cells were cultured in high glucose DMEM high glucose GlutaMAX™ (Invitrogen) supplemented with 10% (v/v) FBS. HeLa cells were from CHO (CHO-C400; not recently authenticated) cells were cultured in DMEM high glucose GlutaMAX™ supplemented with non-essential amino acids and 5% (v/v) FBS. For transfection, 10^6^ cells were seeded in 35 mm dishes and incubated at 37°C overnight. Plasmid cDNA (2 µg) was transfected into HeLa cells using SuperFect (Qiagen) and into U2OS cells using FuGENE™ or Lipofectamine™ 3000. All cell lines were maintained at 37°C and 5% CO_2_ within a Binder CB220 incubator.

### Construction of plasmids encoding modified β and γ subunits

The complete coding region of rat β1 in pcDNA3 (Woods et al., 1996a) was amplified by PCR using primers generating *EcoRI* and *XbaI* ends, and cloned into the *EcoRI*/*XbaI* sites of pcDNA3.1/ZEO to generate β1-ZEO. The coding region of β2 from pcDNA3 (Thornton et al., 1998) was sub-cloned into pcDNA3.1/ZEO to generate *β2-ZEO*. The stop codons in *β1-ZEO* and *β2-ZEO* were mutated into *SnaBI* blunt restriction sites using the QuikChange mutagenesis system (Stratagene). pEYFP-N1 (Clontech) was linearized with *BamHI*, end-filled using T4 polymerase, digested with *EcoRI,* and dephosphorylated with shrimp alkaline phosphatase. *EcoRI-SnaBI* fragments from the β1 and β2 plasmids were gel purified and cloned into pEYFP-N1 to generate *β1-YFP* and *β2-YFP*. Mutations producing G2A substitutions were made in these plasmids using the QuikChange system (Stratagene). All constructs and mutations were confirmed by DNA sequencing. DNA encoding residues 1–268, 1–191, 1–159, 1–124 or 1–106 of the γ2 NTD was amplified by PCR and ligated into the expression vector pEYFP-NI to generate full-length or truncated NTDs with C-terminal YFP tags. Expression of these by transfection in CHO cells, followed by probing of western blots with anti-GFP following SDS-PAGE revealed protein products of the expected size. These plasmids were used for fluorescence microscopy as described below. AMPK-β1 myristoylatable and non-myristoylatable genes were subcloned into pcDNA™5:FRT:TO plasmids using restriction enzymes *KpnI* and *XhoI* for expression in β1/β2 DKO U2OS cells containing FRT sites randomly integrated into their genomes.

### Generation of GBF1-T1337A non-phosphorylatable mutant U2OS cells

CRISPR design was undertaken as follows. A full transcript map of the human GBF1 locus was constructed by combining data from both NCBI [NC_000010.11 (102230643..102382896)] and Ensembl (ENSG00000107862). A nickase guide pair was subsequently identified using the Sanger CRISPR webtool (https://wge.stemcell.sanger.ac.uk/find_crisprs) and chosen on the basis of proximity to residue Thr1337 and low combined off-targeting score, with no off-target sites existing within 1000 bp. The right guide (sense 5′- CCGATGTGGTCAACAGTGGT-3′) and left guide (antisense 5′-GCTGATCGGTGTATCTTGCC-3′) were ordered as 2 nM ALT-R crRNAs from IDT, along with 100 µg Cas9 D10A nickase protein and 20 nM tracrRNA. Asymmetric T1337A single stranded donors (ssODN) were designed for both the upper and lower strands with 90 bp homology upstream and 45 bp downstream of the regions of change; several silent changes were introduced to prevent guide recognition of the insert, to introduce the T1337A mutation and to add a BglII site to facilitate rapid downstream screening. The 181 bp ssODNs were ordered as 4 nM ultramers from IDT.

For electroporation, first, sufficient targeting mixes for two rounds of nucleofection were prepared for each of the ssODNs and transfections performed using a NEPA21 super electroporator (Nepagene). For each transfection, ribonucleoprotein (RNP) was prepared in a 10 µl volume in a sterile 0.2 ml PCR tube consisting of 2.2 µl of 61 µM Cas9 (134 pMol), 1.6 µl of each 50 µM hybridized guide (80 pMol each), 2.2 µl of 100 µM ssODN (220 pMol) and 2.4 µl PBS. A549 cells (European Collection of Cell Cultures) were grown to 70–80% confluency on the day of transfection, trypsinized and washed with Opti-MEM twice to remove all traces of antibiotics. Cells were counted and resuspended to 10^6^ cells per 90 µl using Opti-MEM. Cells (90 µl) were subsequently added to each of the first round 10 µl RNP mixes, transferred to a 2 mM cuvette (Sonidel) and electroporated using a 125 V, 5.0 ms poring pulse. Cells were transferred to 6-well plates containing 2.5 ml prewarmed medium without antibiotics and left to recover overnight. After 18 h, the medium was switched to full medium with penicillin-streptomycin, and cells were expanded to duplicate wells on a 6-well plate. One well was subsequently harvested for genomic DNA (GeneJET Genomic DNA Purification Kit (Thermo scientific, K0722) eluting in 80 µl H_2_O. The other well was expanded to a 10 cm dish to prepare sufficient cells for round 2 of electroporation and to prepare frozen stocks. Cells from round 1 were once again washed in Opti-MEM and retargeted using the duplicate RNP mixes and processed as above.

Screening of pools and single-cell cloning was undertaken as follows. The level of targeting in each of the 4 pools was determined by PCR across GBF1 exon 31 (primers: ex31 F, 5′-TCAGGAACCCAGATTAGCCTGC-3′ and ex31 R, 5′-CTCAAAGTTGTCAGGTGTGATGTGG-3′) using 300 ng genomic DNA and KOD polymerase according to manufacturers' protocols (Sigma-Aldrich, 71085). PCR products were cleaned using PCR clean-up columns (QIAgen PCR purification kit, cat: 28104) and eluted in 50 µl H_2_O; 300 ng of each was digested with *BglII* (Thermo Fisher Scientific, ER0082) and run alongside uncut samples on a 1% TAE agarose gel to determine the level of knock-in. Round 2 of electroporation using the sense strand donor 5′-tgagctcccatcctaccatcagaatgacgtgagcctggatcgagggtacacttccgactcagaggtctacactgaccatggcaggccgggTaaAatCcaTAgatcTgccGcagatgcTgaCgtTgtGaaTTCAggCtggttagtggtgagtgacaatatgggcagcaattgagtctctcct-3′ (lowercase letters are from the native human GBF1 locus, whereas uppercase letters are silent changes introduced to prevent recleavage of the knock-in insert and introduce a BgIII site for screening, as well as the non-silent change to introduce the T1337A mutation) yielded the highest level of editing, with approximately 25% of input PCR product being cleaved via the engineered *BglII* site. This pool was subsequently used for clonal cell deposition and positive clonal lines confirmed by both RFLP and shotgun cloning/sequencing.

### Generation of hygro:Tmem115:3xHA U2OS stable cell line

U2OS cells stably expressing hygro:Tmem115:3xHA were generated using methods reproduced from Abu-Remaileh et al. (2017). Briefly, HEK293T cells (American Type Culture Collection) were plated into 10 cm plates in DMEM high-glucose GlutaMAX™ supplemented with 10% FBS and transfected at ∼60% confluency with 6 μg of pBabeD:hygro:Tmem115:3xHA, 3.8 μg of Clontech GAG/POL, and 2.2 μg of Clontech VSVG in Opti-MEM™ reduced serum medium using Lipofectamine™ 3000 at 37°C and 5% CO_2_ for 5 h. The medium was replaced with DMEM high-glucose GlutaMAX™ supplemented with 10% FBS overnight and then refreshed the following morning. After 24 h, the virus was harvested in this medium and filtered using a 0.45 μm polyethersulphone sterile syringe filter (LLES). Fresh medium was applied, and a second harvest of virus was collected after another 24 h. U2OS cells in 10 cm plates and at ∼60% confluency were transduced with pBabeD:hygro:Tmem115:3xHA using Polybrene (10 μg/ml) over 24 h and then placed under hygromycin selection and single-cell sorted using flow cytometry. Clones were screened from 6 cm plates using anti-HA antibody.

### Affinity purification of Golgi vesicles from hygro:Tmem115:3xHA U2OS cells

Affinity purification was performed using the Miltenyi Biotec µMACS^TM^ Isolation Kit, as briefly described below. Approximately 10^7^ cells were grown in 15 cm plates and scraped, and the packed cell volume (PCV) was measured. Cells were re-suspended in 1 ml of 0.25 M sucrose solution per 1 ml of cells and homogenized on ice. The homogenate was centrifuged at 3000 ***g*** for 15 min at 2–8°C, transferred into a fresh tube, and its protein concentration measured. 50 µl of anti-HA MicroBeads were added to 200 µg protein of each lysate, and mixed gently by pipetting. Beads and lysates were incubated on a rotator at room temperature (RT) for 30 min. µColumns were placed in the magnetic field of the µMACS magnetic bead separating stand and the lysate containing beads was applied. µColumns were washed using four time with 200 µl of Wash Buffer 1 followed by once with 100 µl of Wash Buffer 2 before applying 20 µl of pre-heated (95°C) Elution Buffer for 5 min, followed by elution using 50 µl of pre-heated (95°C) Elution Buffer. The eluents were collected into 1.5 ml Eppendorf tubes and analysed by immunoblotting.

### Generation of β1 and β2 WT and G2A U2OS cell lines

AMPK-β1:WT, AMPK-β1:G2A, AMPK-β2:WT, and AMPK-β2:G2A genes were stably inserted into U2OS FRT Flp-In T-Rex cells using the inducible expression vector pcDNA™5/FRT, containing tandem TetO sequences and zeocin resistance, alongside the pOG44 Flp-recombinase expression vector. U2OS FRT Flp-In T-REx DKO cells cultured in 6 cm plates in DMEM high-glucose GlutaMAX™ serum-free medium were transfected at ∼60% confluency with 3 ng pOG44 and 0.3 ng pcDNA™5:FRT:TO AMPK-β vector (ratio of 9:1) using 10 μl of FuGENE™. The modified genes of the AMPK-β subunit inserted into the genomic FRT site via homologous recombination with the vector FRT site. Cells were incubated at 37°C and 5% CO_2_ for 48 h, trypsinized and cultured in DMEM high glucose GlutaMAX™ supplemented with 10% FBS. Hygromycin (200 μg/ml) was added after 24 h to select for insertion of the pcDNA™5:FRT:TO AMPK-β vector. Expression of the modified AMPK-β subunits required the Tet-on system, where cells were treated with 0.01, 0.1, 1.0 or 10.0 μg/ml of tetracycline or vehicle (ethanol) over 24, 48 and 72 h and expression of the AMPK-β1 or -β2 subunit was measured and quantified relative to endogenous expression using AMPK-β1 or -β2 specific antibodies. The optimal tetracycline treatment to equalize transfected and endogenous expression of AMPK-β1 and -β2 subunits was 0.01 μg/ml over 24 h.

### Immunoblotting

Cells lysis was performed on ice using 20 mM Tris-HCl lysis buffer pH 7.5 containing 50 mM NaCl, 2.5 mM sodium pyrophosphate, 1 mM Na orthovanadate, 1 mM EDTA, 1 mM EGTA, 1 mM DTT, 0.1 mM benzamidine, 0.1 mM PMSF, 5 μg/ml soybean trypsin inhibitor and 1% (v/v) Triton X-100, after washing cells with ice-cold PBS. Proteins were analysed by electrophoresis using the Novex^TM^ Midi-Cell system (Invitrogen) on 4–12% Bis-Tris SDS-polyacrylamide gels (Invitrogen), except for analysis of ACC where 3–8% Tris-acetate gels were used. Proteins were electrophoretically transferred onto 0.45 μm nitrocellulose membranes using a BIO-RAD iBlot2™ apparatus, blocked by incubation with Li-Cor Intercept™ (PBS) blocking buffer for 60 min, and probed with affinity-purified sheep, mouse or rabbit antibodies (0.2–1 mg/ml). Membranes were then either probed with protein G coupled to horseradish peroxidase (0.2 mg/ml, Sigma-Aldrich) and detected using enhanced chemiluminescence (Amersham Pharmacia) or by Alexa Fluor secondary fluorescently tagged antibodies using the LI-COR IR imager or LI-COR Odyssey™ CLx, with densitometry performed using Image Studio Lite^TM^ version 5.2. Full uncropped images for western blots shown in the paper are given in Fig. S9.

### VSVG3–GFP trafficking

VSVG–GFP was transiently transfected into U2OS cells plated in a 24-well plate using 2 μg of FuGENE® Transfection Reagent in a 3:1 ratio to VSVG–GFP DNA. After incubation for 15 min at RT, the mixture was made up to a final volume of 100 μl using serum-free MEM and incubated for a further 5 min at RT. The final mixture was added dropwise to each well and incubated for 24 h at 37°C and 5% CO_2_. All conditions were incubated at the restrictive temperature of 40°C for 6 h and compounds under test were applied 60 min prior to either being moved to the incubator set to the permissive temperature of 32°C or remaining in the 40°C incubator for 0, 30, 90 or 240 min.

### Deconvolution microscopy

Deconvolution microscopy was performed using a DeltaVision Restoration Microscope (Applied Precision Inc, Issaquah, WA, USA) built around a Nikon Inverted microscope fitted with a 100×/1.4 NA PlanApo lens. Images were acquired using a Roper Scientific Interline cooled CCD camera (5 MHz Micromax 1300YHS) and optical *Z* sections were captured at either 0.2 µm (fixed cells) or 0.4 µm intervals (live cells) to encompass the whole cell. 3D data sets were deconvolved using the SoftWoRx software (Applied Precision Inc.) running on a Silicon Graphics Octane workstation. 2D maximum intensity projections of 3D data sets were generated using SoftWoRx. TIFFs and movies were captured using Mediarecorder (Silicon Graphics Inc.) and processed for publication in Adobe Photoshop. Quantitative analysis of intracellular fluorescence signals was performed using 2D polygon and 3D modelling tools within SoftWoRx.

### Preparation of fixed cells for deconvolution microscopy

HeLa or CHO cells (10^6^ cells) were seeded into 35 mm dishes containing 2×10 mm diameter glass coverslips and incubated at 37°C overnight. Cells were transfected with cDNAs encoding combinations of AMPK heterotrimer complexes (2 µg per subunit). Cells were fixed 24 h post transfection for 20 min at RT in PBS containing 4% (w/v) paraformaldehyde. Excess fixative was quenched in PBS containing 100 mM glycine (15 min), and cells were permeabilized in PBS containing 0.1% (v/v) Triton X-100 (15 min). Coverslips were incubated in PBS containing 5% (v/v) donkey serum (Jackson Laboratories) to block non-specific binding, rinsed in PBS and incubated at 37°C for 1 h in PBS containing 1% (v/v) donkey serum and appropriate primary antibody (3–10 µg/ml). They were washed extensively in PBS and then incubated at 37°C for 1 h in PBS containing 1% (v/v) donkey serum and a secondary antibody conjugated to either FITC or Texas Red (8 µg/ml). Coverslips were washed extensively in PBS and DNA and nuclei stained by incubation in PBS containing 1 µg/ml DAPI. They were then mounted in either 0.5% *p*-phenylenediamine in 20 mM Tris-HCl pH 8.8 and 90% glycerol and viewed using the DeltaVision microscope.

### Confocal microscopy

Immunofluorescence confocal microscopy was performed using the LSM Zeiss Xenon 710 fluorescence confocal microscope. The pinhole aperture used was 1 Airy unit, objective 60×1.5 NA and immersion oil 1.514. Images were taken as optical sections using an optical slice thickness of 1 μm or as a *Z*-stack using eight *Z*-sections collected at 0.33 μm. The latter was only used when colocalization analysis was not performed. All images were taken using 100×100 pixels and were processed using ImageJ™ and saved as tiff files. Each condition was performed in duplicate, each experiment performed twice and ten random images were taken from each coverslip. Intensity and colocalization (Pearson's correlation co-efficient) analysis was performed using Volocity™. Intensity (arbitrary units) was determined by using a measurement protocol that included the ‘find objects’ command to determine the region of interest (ROI) of the relevant organelle, the ‘calculate object colocalization’ command to measure the overlap between the ROI and VSVG–GFP, and the ‘calculate thresholds’ command to measure the intensity of the overlap between the ROI and the GFP.

### Preparation of fixed cells for confocal microscopy

Sterilized no. 1.5 glass cover slips (13 mm diameter) were individually placed in each well of a 24-well plate using conditions. U2OS cells were plated at ∼30% confluency in 1 ml of DMEM high-glucose GlutaMax™ medium. After the application of treatments, cells were fixed using 0.5 ml of PBS containing 4% (w/v) formaldehyde for 15 min at RT, washed using PBS containing 0.2% (w/v) BSA, permeabilized with PBS containing 1% (v/v) Triton-X 100 for 10 min at RT and blocked using PBS containing 1% (w/v) BSA for 60 min at RT. Cells were incubated with primary antibody prepared in PBS containing 0.2% (w/v) BSA for 16 h at 4°C. Glass slips were washed for thre times for 5 min using PBS containing 0.2% (w/v) BSA, and pre- and post-incubation with Alexa Fluor^TM^ conjugated secondary antibodies prepared in PBS containing 0.2% (w/v) BSA were added followed by incubation at RT for 60 min in the dark. DAPI was prepared in PBS containing 0.2% (w/v) BSA sodium azide and 1 μg/ml was added to each well for 10 min at RT in the dark. Glass slips were mounted onto glass slides using ProLong™ Glass Antifade Mountant and sealed using clear nail polish. Slides were stored at −20°C.

### Quantification of Golgi morphology

Golgi morphology was analysed using a macro created in Fiji ImageJ Software™ by Dr Graeme Ball, Dundee Imaging Facility Specialist, Centre for Advanced Specific Technologies, University of Dundee. Raw LSM image files were used for analysis. The macro applied a filter to suppress noise and analysed particles to count and measure Golgi fragments by selecting the channel of interest.

Macro details are:

run(”Median...”, “radius=2”);

setAutoThreshold(”MaxEntropy dark no-reset”);

run(”Set Measurements...”, “area mean standard min centroid display redirect=None decimal=3”);

run(”Analyze Particles...”, “size=0.05-Infinity show=Overlay display exclude summarize”);

### Statistical analysis

Statistical analysis was performed using GraphPad Prism 5 for Mac, using unpaired two-tailed *t*-tests for two comparisons, or one- or two-way ANOVA with the Holm–Sidak multiple comparison test for more than two comparisons. Unless indicated otherwise, *n* values are for six biological replicates (separate dishes of cultured cells), from each of which we first measured the mean of five technical replicates (individual cells within one field). In Figures, *P* values are indicated as follows: *P<0.05, **P<0.01, ***P<0.001, ****P<0.0001.

### Reagents and code availability

Reagents and cell lines not commercially available may be requested from the corresponding author. For software see the ‘Quantification of Golgi Morphology’ above.

## Supplementary Material

10.1242/joces.262182_sup1 Supplementary information
