# Supplementary material for: AMPK associates with and causes fragmentation of the Golgi by phosphorylating the guanine nucleotide exchange factor GBF1
Source: J Cell Sci. 2024 Dec 23;137(24):jcs262182. doi: 10.1242/jcs.262182 (PMC11827860; doi:10.1242/jcs.262182)
Supplement: Supplementary information [file joces-137-262182-s1.pdf]

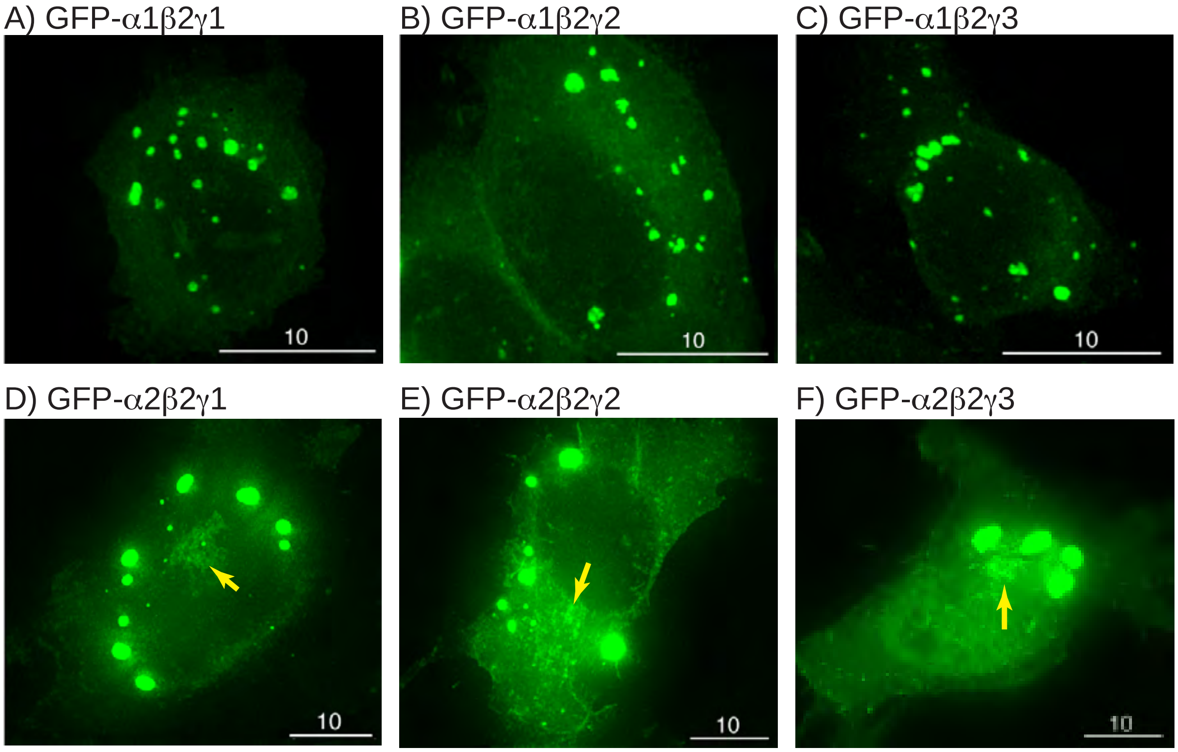

Fig. S1. Targetting of β2 complexes to different subcellular locations. Images show fluorescence micrographs obtained by deconvolution microscopy (0.2 µm optical sections) of transfected HeLa cells co-expressing β2 with GFP-α1 (A-C, top panels) or GFP-α2 (D-F, bottom panels), and γ1 (A, D), γ2 (B, E) or γ3 (C, F). The enrichment of fluorescence adjacent to one pole of the nucleus is indicated by yellow arrows in the bottom panels. All scale bars are 10 µm.

Journal of Cell Science • Supplementary information

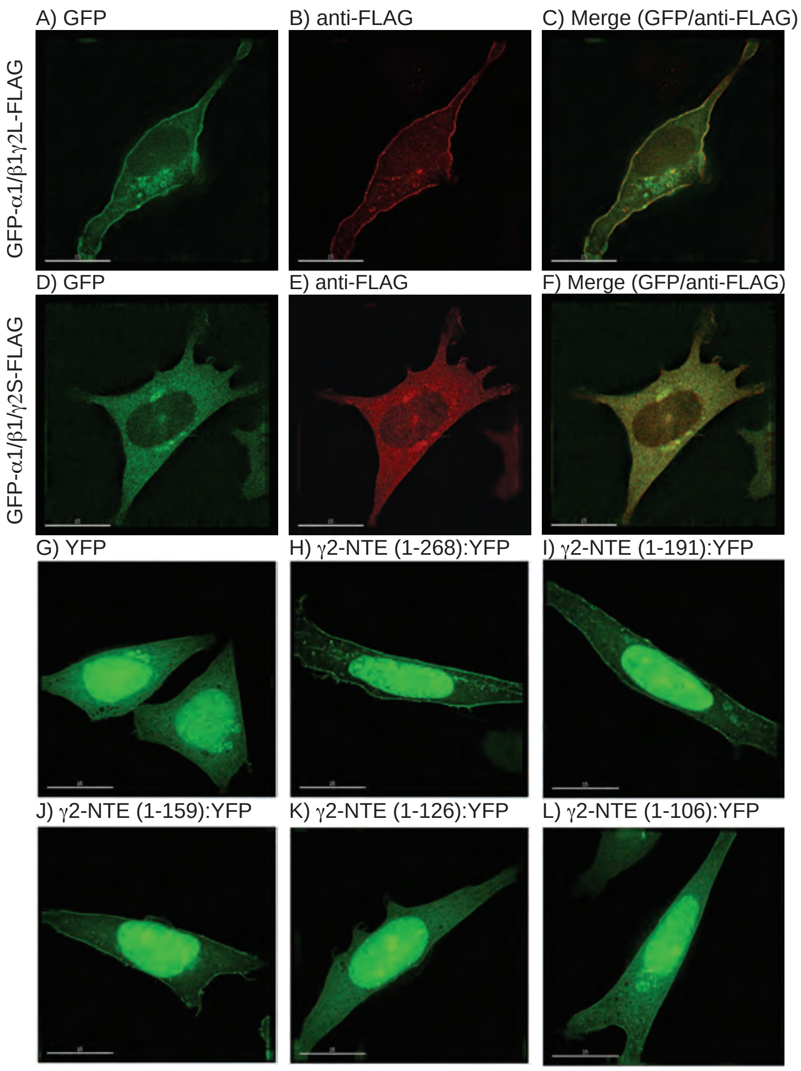

Fig. S2. Targetting of γ2 complexes to the cell margin. Images in A-F are optical sections of CHO cells co-expressing GFP-α1 and β1 with FLAG-tagged full-length γ2 (γ2L, A-C) or an N-terminal truncation (γ2S, D-F). Images in G-L are optical sections of CHO cells expressing YFP (G), the γ2 N-terminal extension (NTE, 1-268) fused to YFP (H), or the indicated truncation of the γ2 NTE fused to YFP. A and D show GFP, B and E show Texas Red-labelled anti-FLAG and C and F are merged images of A/B and D/E respectively. Figure were obtained by deconvolution microscopy. Scale bars are 15 µm.

Journal of Cell Science • Supplementary information

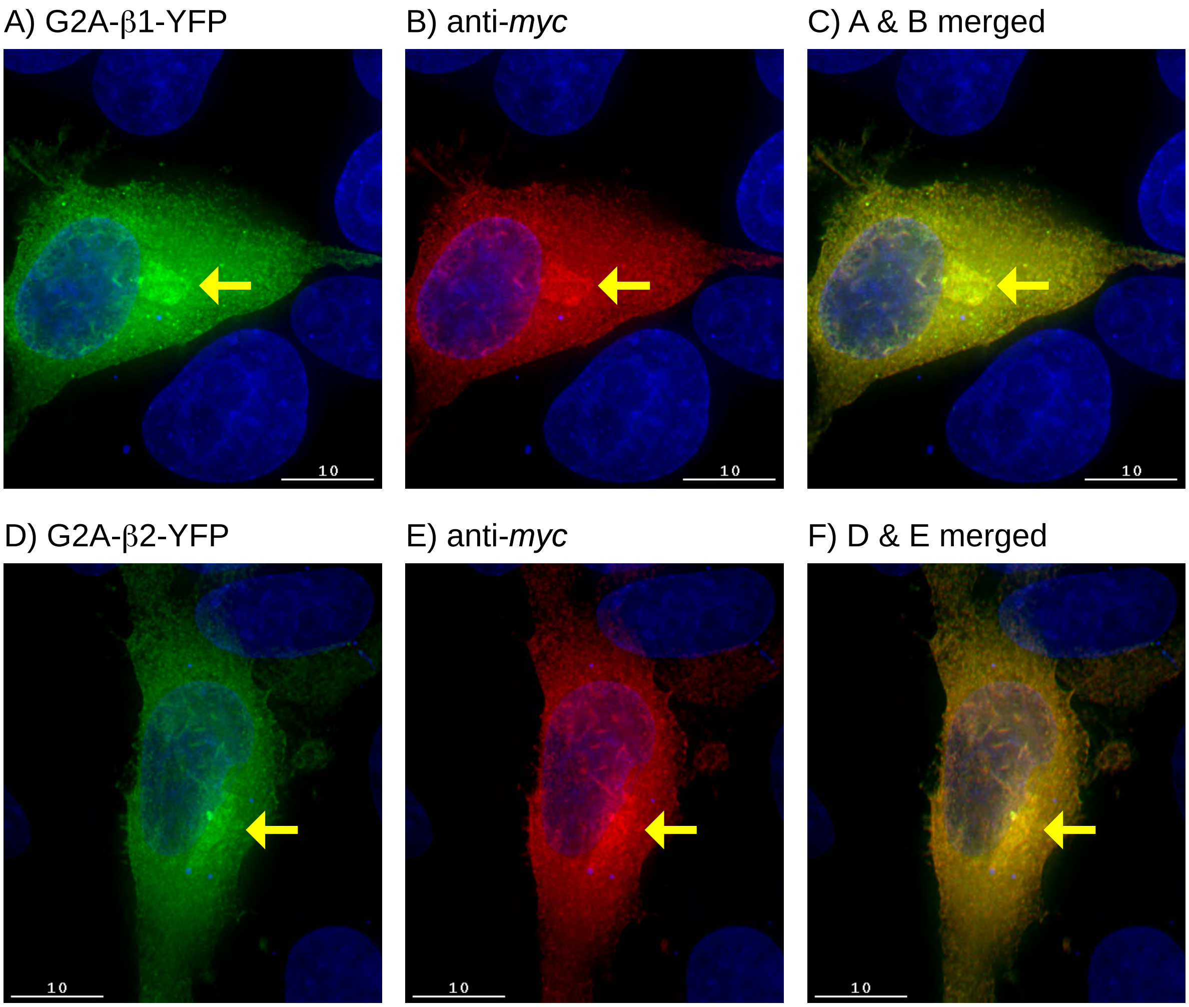

Fig. S3. Non-myristoylated β1 and β2 mutants are also enriched, together with α1, at the Golgi apparatus. Images show fluorescence micro-graphs obtained by deconvolution microscopy (whole cell projections) of transfected HeLa cells co-expressing G2A mutants of β1-YFP (A-C) or β2-YFP (D-E) with myc-α1 and γ1. The enrichment of fluorescence adja-cent to one pole of the nucleus is still evident with the G2A mutants (yel-low arrows). All scale bars are 10 μm.

Journal of Cell Science • Supplementary information

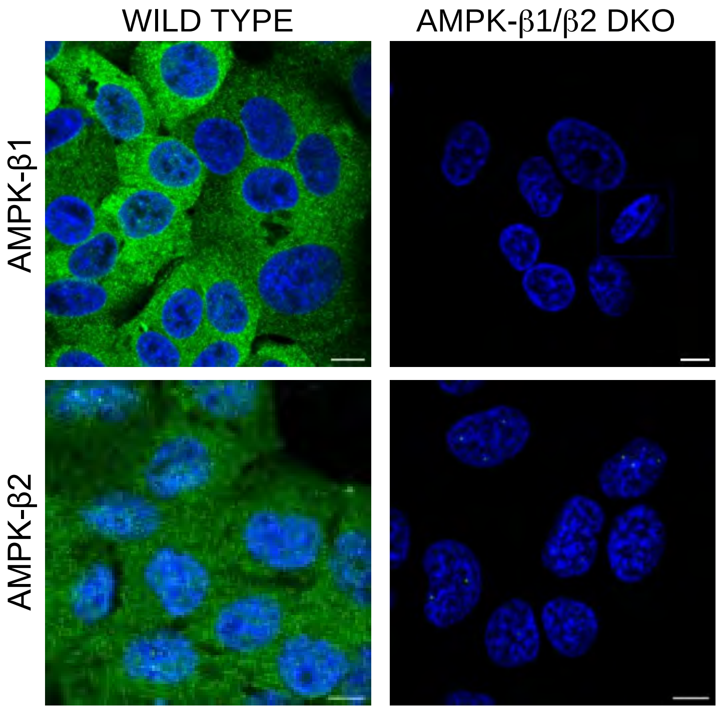

Fig. S4. Wild type and double AMPK-β1/-β2 knockout U2OS cells were fixed and stained with 4′,6-diamidino-2-phenylindole (DAPI, blue) to label the nuclei, and with rabbit anti-β1 (AbCam ab32112) or anti-β2 (AbCam ab24472) detected using goat anti-rabbit antibody (green). Images were obtained by confocal microscopy. The scale bars are 10 μm.

Journal of Cell Science • Supplementary information

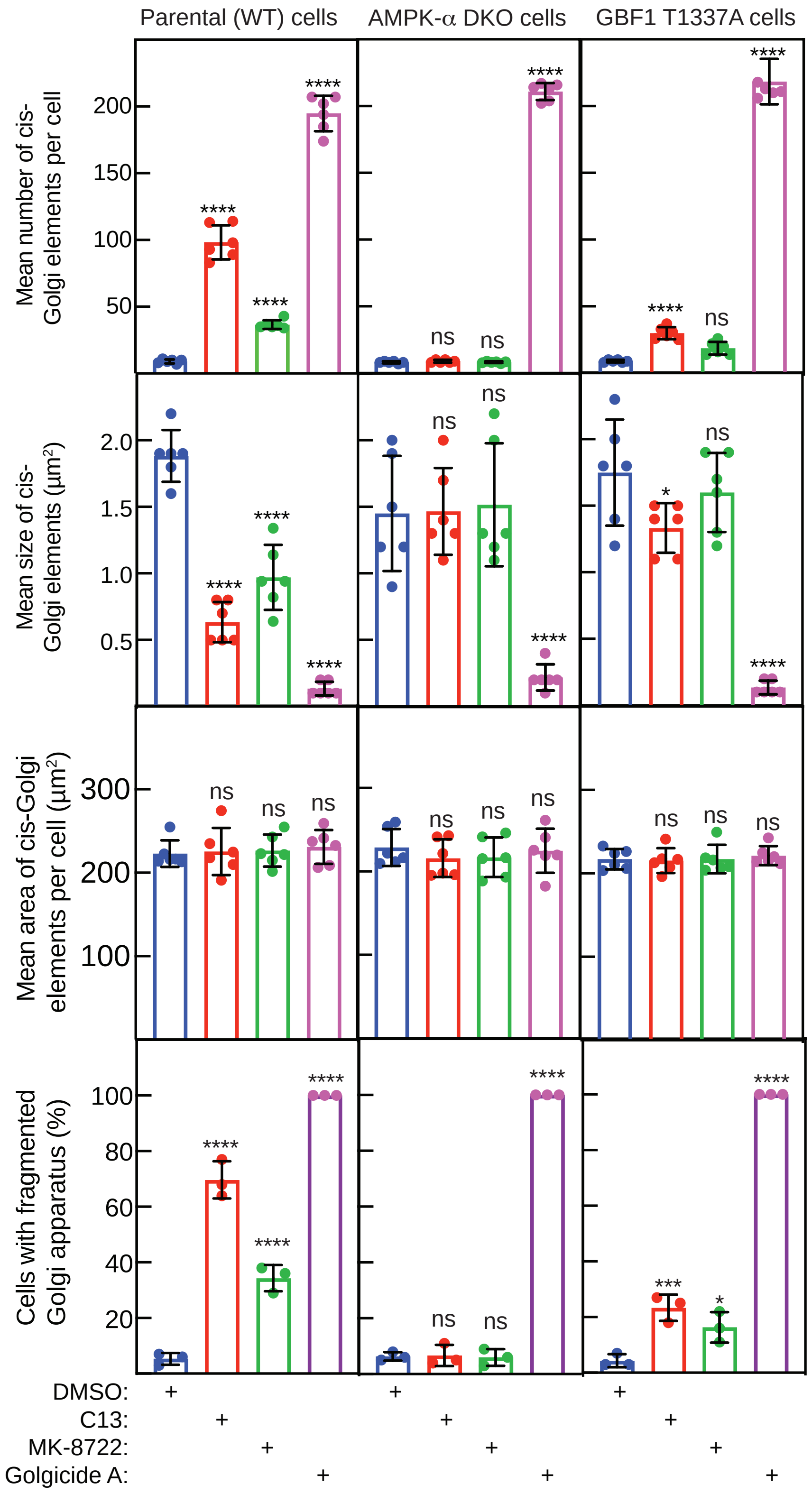

Fig. S5. Q**uantification of results from Fig. 4B, showing effects of AMPK activators and Golgicide A on Golgi structure.** In the top 3 panels, results are means ± SD for 6 biological replicates, and in the bottom panels are means ± SD for 3 biological replicates. Mean values significantly different from DMSO controls (1-way ANOVA, Holm-Sidak post test) are indicated: *P<0.05, **P<0.01, ***P<0.001, ****P<0.0001; ns, not significant. Results in the top three panels were determined direct from cell images in an unbiased manner using ImageJ software, while those in the bottom panels were obtained by visual inspection of images.

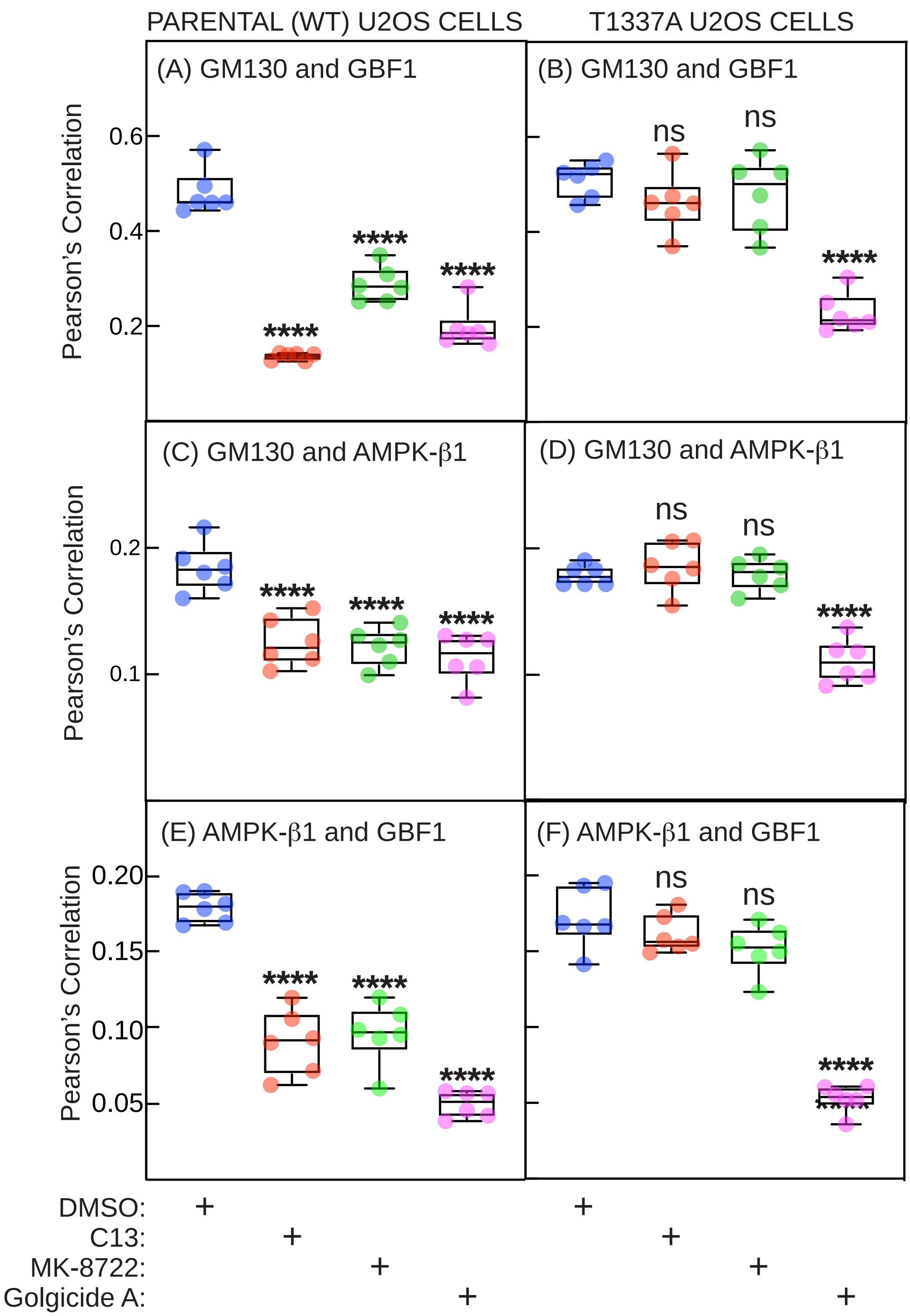

Fig. S6. Quantification of IFM images from Fig. 5. The results show changes in Pearson's correlations (Boxes: mean, upper and lower quartiles; Whiskers: minimum and maximum, every point shown, n = 6) after treatment for 1 hr with DMSO (0.1%, control), C13 (300 µM), MK-8722 (200 nM) or Golgicide A (1 µM) in parental (WT) U2OS cells (left) or T1337A knock-in cells (right). Mean values significantly different from DMSO controls (1-way ANOVA, Holm-Sidak post test) are indicated: *P<0.05, **P<0.01, ***P<0.001, ****P<0.0001; ns, not significant.

Journal of Cell Science • Supplementary information

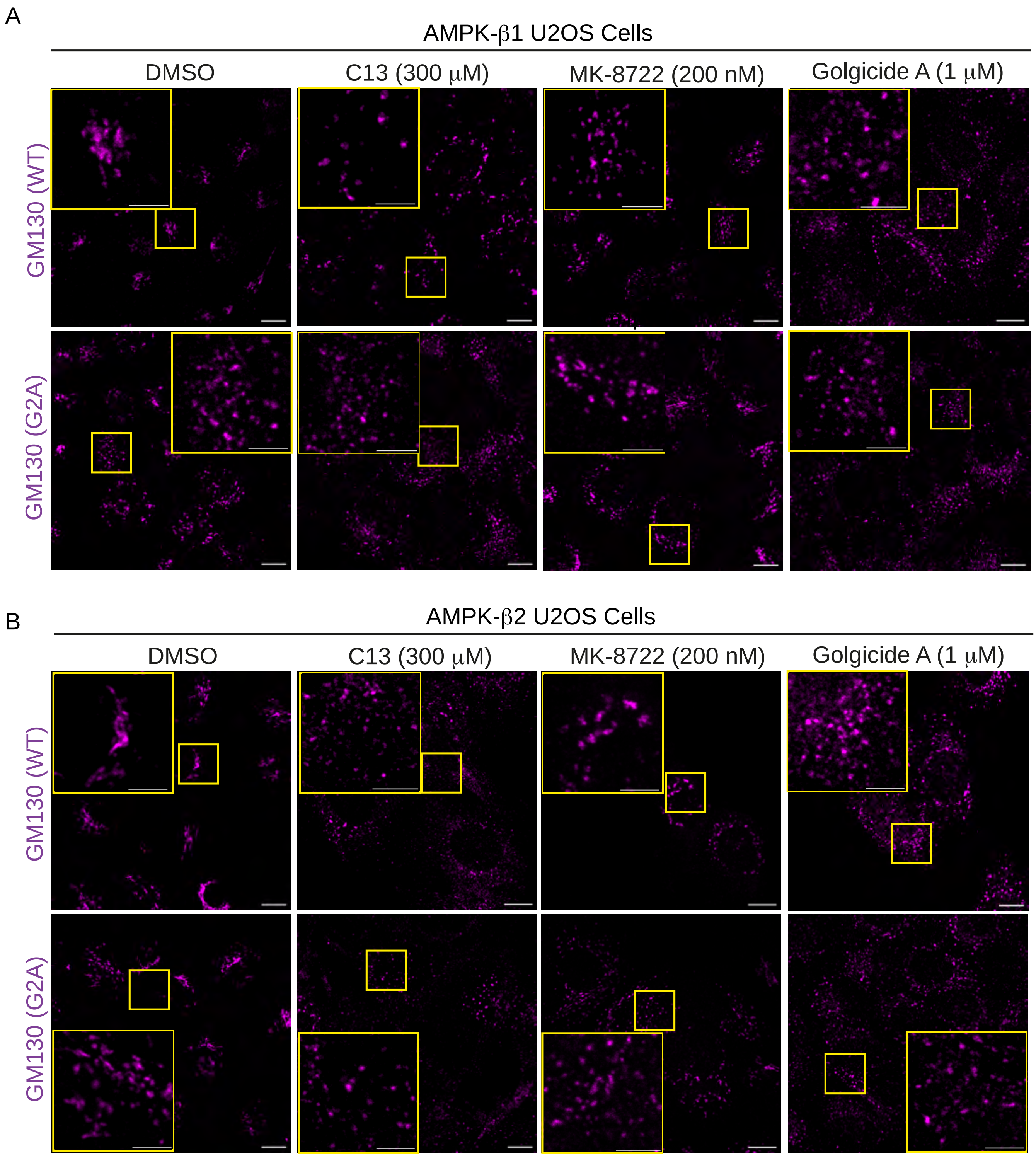

Fig. S7. Fragmentation of the *cis*-Golgi (GM130 marker) in response to C13, MK-8722 or Golgicide A in cells expressing wild type or non-myristoylatable (G2A) mutants of AMPK-β1 or -β2. WT or G2A mutants of AMPK-β1 (top) or AMPK-β2 (bottom) were stably expressed from the single Flp recombinase target site in double knockout (AMPK-$\beta1^{-/-}$/-$\beta2^{-/-}$) U2OS cells. Cells were treated with DMSO, C13, MK-8722 or Golgicide A for 1 hr, fixed, and stained for IFM with anti-GM130 antibodies. Images were obtained by confocal microscopy. The inset areas within the large yellow rectan-gles are the areas shown with small yellow rectangles at higher magnification. The scale bars are 10 µm for the main images and 5 µm for the insets.

Journal of Cell Science • Supplementary information

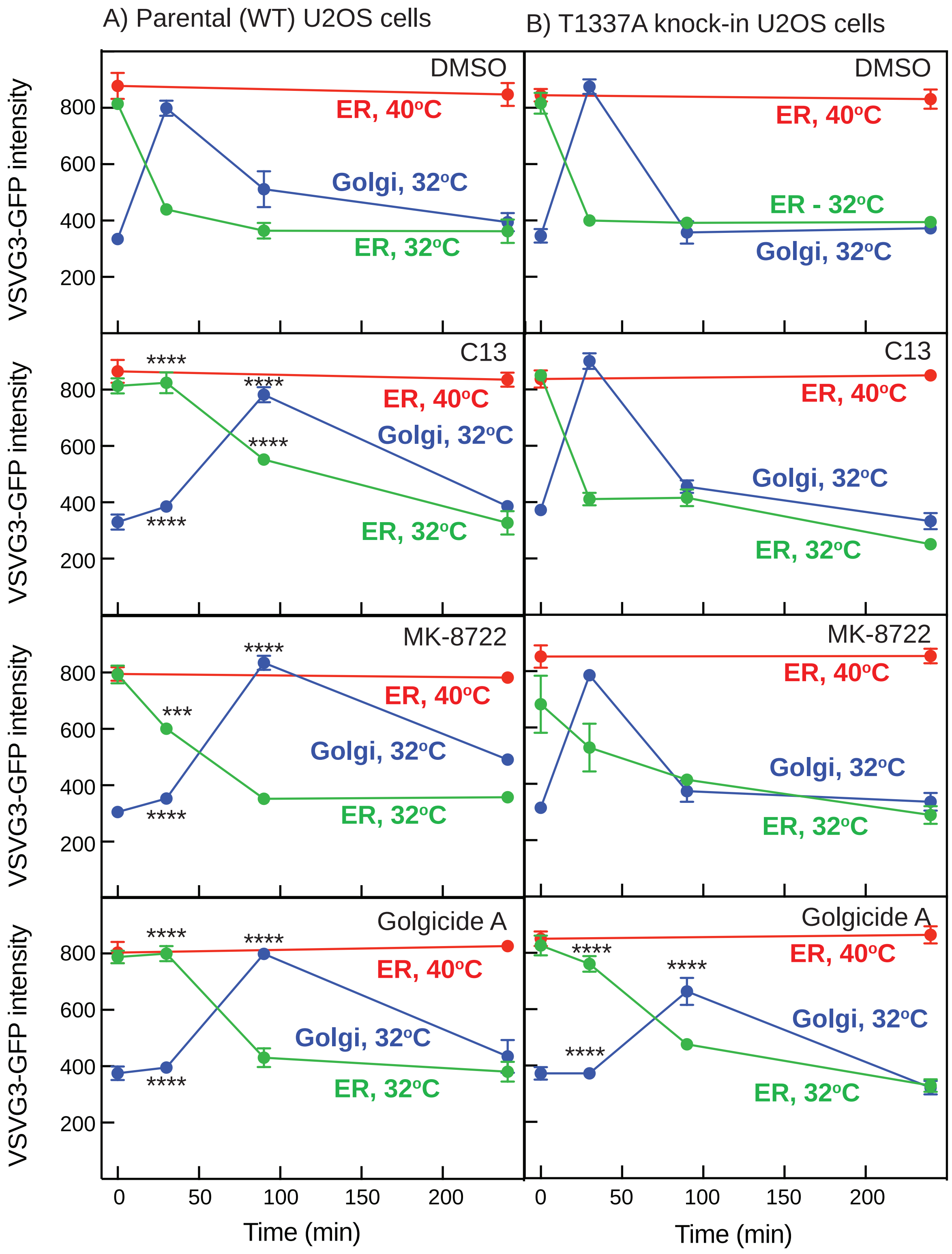

Fig. S8. Transit of a temperature-sensitive viral membrane protein (VSVG) from the ER to the Golgi is delayed by AMPK activation. DNA encoding VSVG3-GFP was transfected into the cells for 24 hr at the restrictive temperature (40°C), when the protein misfolds and is retained in the ER. On transfer to the permissive temperature (32°C), the protein folds and transits from the ER to the Golgi and then to the plasma membrane. The results (mean ± SEM, n = 6) represent quantification of results of which Fig. 8 is an example. For each image, the intensity of VSVG-GFP fluorescence in a cell area positive for an ER marker (calnexin) or a Golgi marker (GM130) was quantified. Asterisks show mean values that are significantly different by 2-way ANOVA (Holm-Sidak post test) from DMSO controls at the same time point. Graphs on the left (A) are from cells with WT GBF1 and on the right (B) from T1337A GBF1 knock-in cells. Also shown (red lines) is that VSV3G3:GFP is retained in the ER at 40°C in both cell types.

Journal of Cell Science • Supplementary information

Figure 4:

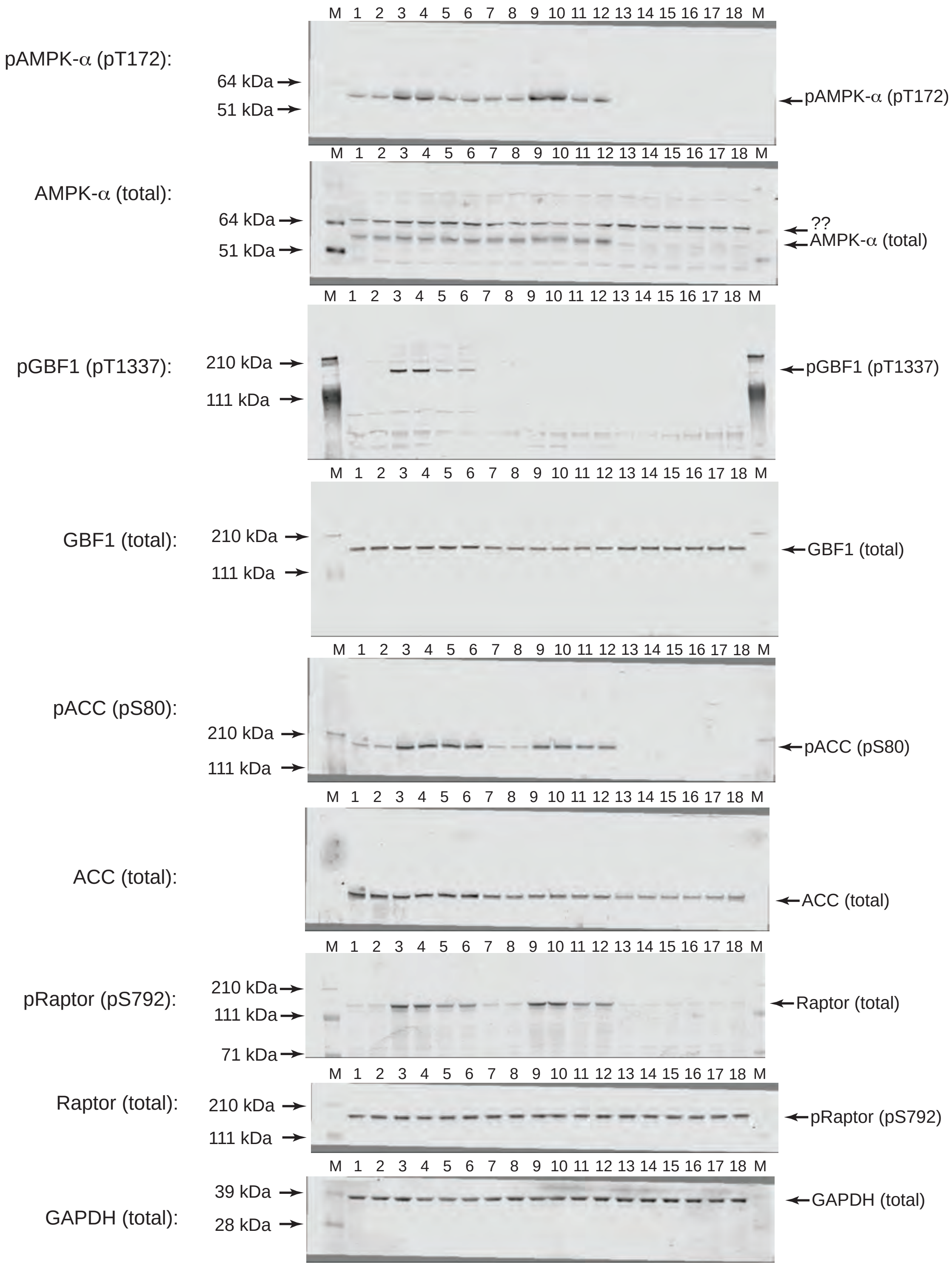

NOTES:

- Molecular masses given for markers are nominal values quoted by suppliers for the gel system used, and are approximate values only.
- Bands labelled “??” are unknown proteins that are detected non-specifically by the antibodies used.
- The finding that the band labelled “pGBF1 (pT1337)” in the anti-pGBF1 blot disappears in the T1337A knock-in cells (lanes 7-12) and co-migrates with GBF1 (total) in the anti-GBF1 blot confirms that the anti-pGBF1 antibody is specific for phosphorylated Thr1337.
- Note that pGBF1/GBF1 migrate just ahead of the 210 kDa marker (expected mass is 207 kDa).
- The finding that the band labelled “pAMPK-α (pT172)” in the anti-pAMPK-α blot disappears in the AMPK-α1/-α2 DKO cells (lanes 13-18) and co-migrates with AMPK-α (total) in the anti-AMPK-α blot confirms that the anti-AMPK-α antibody is specific for phosphorylated Thr172.
- Note that AMPK-α migrates between the 64 and 51 kDa markers (expected mass is 63 kDa).
- Total ACC was detected using streptavidin, which does not detect the markers.

Journal of Cell Science • Supplementary information

Figure 6:

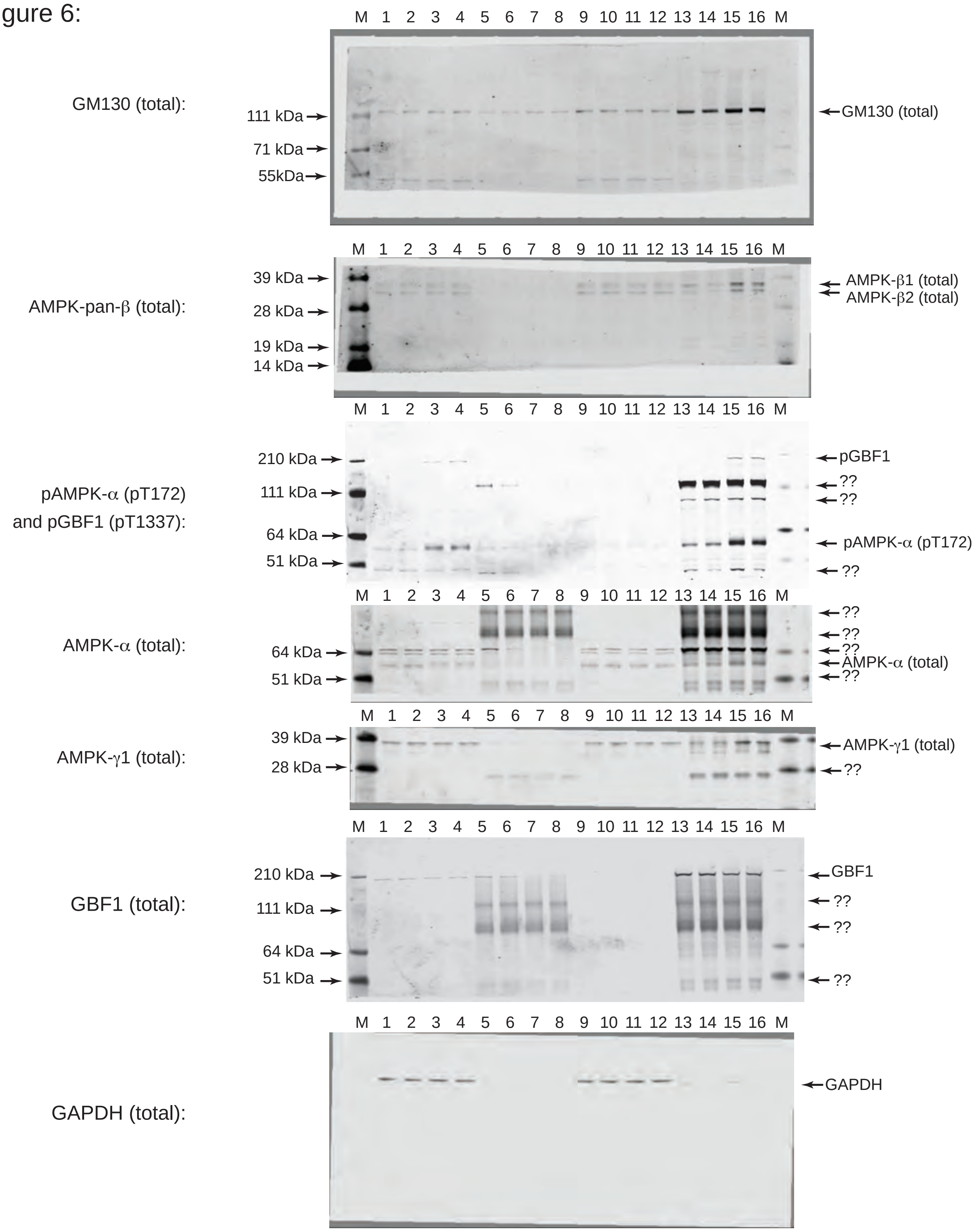

NOTES:

- Molecular masses given for markers are nominal values quoted by suppliers for the gel system used, and are approximate values only.
- Bands labelled "??" are unknown proteins that are detected non-specifically by the antibodies used - some of these appear to be abundant proteins of the Golgi.
- Due to limited availability of protein in the purified Golgi membranes, the blot probed with anti-pAMPK-α was also probed with anti-pGBF1. This approach was possible because AMPK-α and GBF1 run at markedly different positions in the gel. These antibodies had already been validated for Western blotting, and the mobility of the AMPK-α and GBF1 bands relative to the markers established, in Fig. 4. We are therefore confident that the AMPK-α and GBF1 bands have been identified correctly.

Journal of Cell Science • Supplementary information

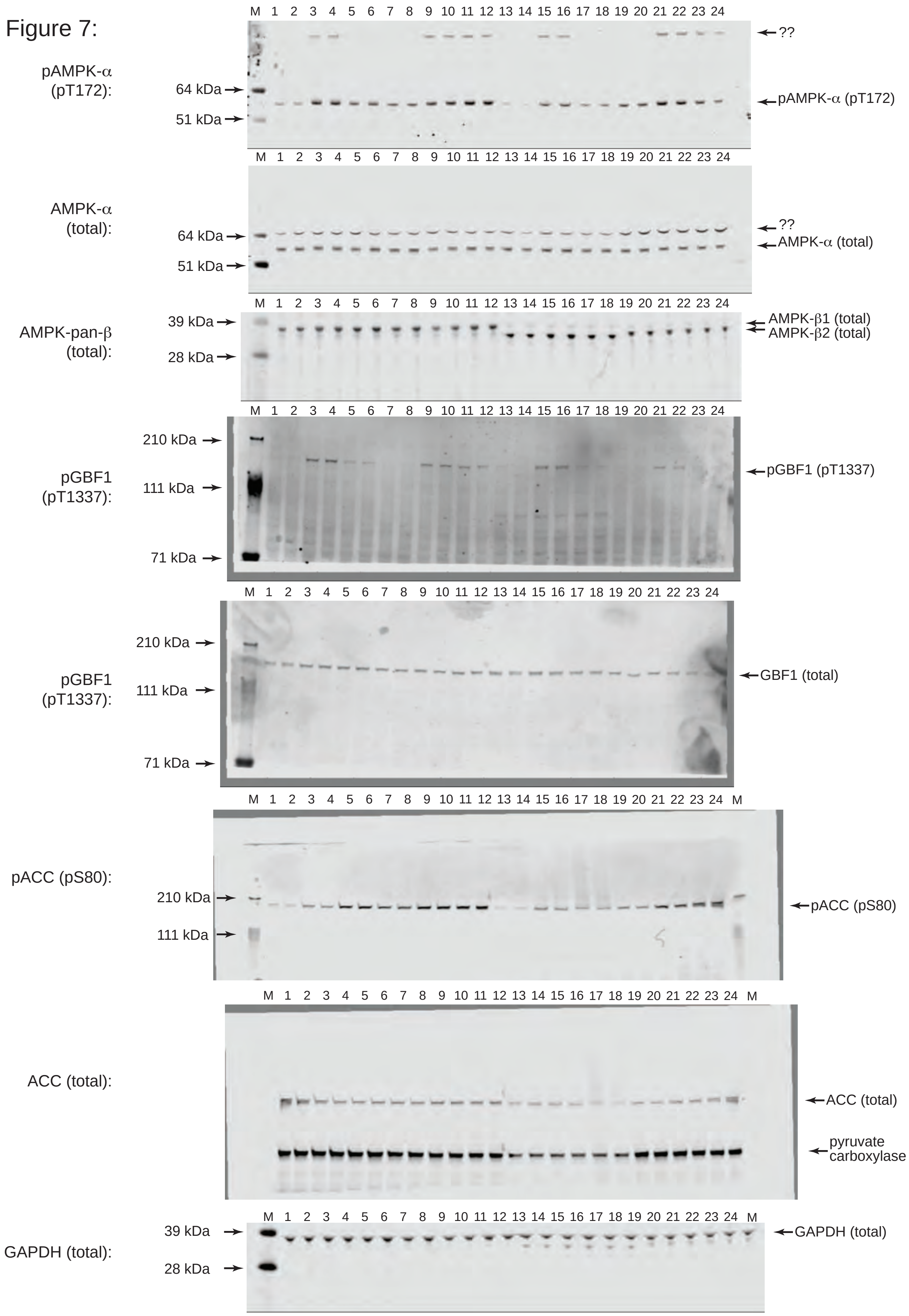

NOTES:

- Total ACC was detected using streptavidin, which does not detect the markers but does detect other biotin-containing enzymes including pyruvate carboxylase.

Fig. S9. Blot transparency.

Journal of Cell Science • Supplementary information

Table S1. Commercial Primary Antibodies.

Antibody	Vendor	RRID	Species	Application	Concn/dilution
ACC pSer79	Cell Signaling Technology	AB_330337	Mouse	Blotting	1:1000
ACBD3	Merck	AB_2220068	Mouse	Blotting<br>Microscopy	1.0 µg/mL<br>2.0 µg/mL
AMPK-α1/α2	Abcam	AB_1603618	Mouse	Blotting	1.0 µg/mL
AMPK-α1/α2 (pThr172)	Cell Signaling Technology	AB_331250	Rabbit	Blotting	1:1000
AMPK-β1/β2	Cell Signalling Technology	AB_10828832	Rabbit	Blotting	1:1000
AMPK-β1	Abcam	AB_722767	Rabbit	Blotting<br>Microscopy	1.7 µg/mL<br>1.7 µg/mL
AMPK-β2	Abcam	AB_2893182	Rabbit	Blotting<br>Microscopy	0.8 µg/mL<br>1.6 µg/mL
AMPK-γ1	Proteintech	AB_2882478	Mouse	Blotting	1.0 µg/mL
Calnexin	Abcam	AB_2069006	Rabbit	Blotting<br>Microscopy	1.0 µg/mL<br>2.0 µg/mL
GAPDH	Sigma	AB_2107445	Mouse	Blotting	1.0 µg/mL
GBF1 (pT1337)	Immuno-Biological Labs, Inc.	AB_10705713	Rabbit	Blotting	1.0 µg/mL
GBF1	BD Biosciences	AB_10705713	Mouse	Blotting<br>Microscopy	1.0 µg/mL<br>2.0 µg/mL
GM130	Abcam	AB_880266	Rabbit	Blotting<br>Microscopy	0.6 µg/mL<br>1.2 µg/mL
HA	Cell Signalling Technology	AB_10691311	Mouse	Blotting	1.0 µg/mL
Myc	CST	AB_331783	Mouse	Microscopy	1:1000
pRaptor (pSer792)	Cell Signalling Technology	AB_2934061	Rabbit	Blotting	1:1000
Raptor	Cell Signalling Technology	AB_561245	Rabbit	Blotting	1:1000
Tmem192	Abcam	AB_3095683	Rabbit	Blotting, Microscopy	2.5 µg/mL

Table S2. Commercial Secondary Antibodies and streptavidin.

Antibody/label	Vendor	RRID	Species	Channel	Concn/dilution
Anti-rabbit IgG/ Alexa Fluor	ThermoFisher	AB_2576217	Donkey	488	2.0 µg/mL
Anti-rabbit IgG/ Alexa Fluor	ThermoFisher	AB_141637	Donkey	594	2.0 µg/mL
Anti-mouse IgG/ Alexa Fluor	ThermoFisher	AB_2534088	Goat	488	2.0 µg/mL
Anti-mouse IgG/ Alexa Fluor™	ThermoFisher	AB_2534091	Goat	594	2.0 µg/mL
Anti-rabbit IgG/ IRDye®	Li-COR	AB_621843	Goat	800	1:10,000
Anti-mouse IgG/ IRDye®	Li-COR	AB_10956588	Goat	700	1:10,000
Streptavidin DyLight™ 800	ThermoFisher			800	1:1000

Journal of Cell Science • Supplementary information
